# Comparative Genomic and Transcriptomic Analyses of ^60^Co-Mutagenized *Scheffersomyces stipitis* Strains: Identification of Candidate Genes Associated with High-Ethanol-Producing Xylose-to-Ethanol Fermentation

**DOI:** 10.3390/life16081307

**Published:** 2026-08-10

**Authors:** Hao Zou, Yuanjie Zhou, Suiyin Lin, Tingting Pang, Linxi Zhang, Jing Zhou, Renzhi Wu

**Affiliations:** 1 Guangxi Key Laboratory of Polysaccharide Materials and Modification, Guangxi-Indonesia Joint Laboratory for Microbial Resources and Artificial Intelligence, School of Marine Sciences and Biotechnology, Guangxi Minzu University, Nanning 530008, China; 15680002475@163.com (H.Z.);; 2 National Key Laboratory of Non-Food Biomass Technology, National Engineering Research Center for Non-Food Biorefinery, Guangxi Academy of Sciences, Nanning 530007, China

**Keywords:** *Scheffersomyces stipitis*, xylose fermentation, comparative genomics, transcriptomics, cellulosic ethanol, strain improvement

## Abstract

Sugarcane bagasse is an important renewable lignocellulosic resource, yet its bioconversion efficiency remains low, primarily because wild-type Saccharomyces cerevisiae cannot utilize xylose, which limits the industrial production of cellulosic ethanol. In this study, a high-ethanol-yielding strain 31.1 was obtained from *Scheffersomyces stipitis* (formerly known as *Pichia stipitis*) 1960 through ^60^Co mutagenesis and long-term domestication. Strain 31.1 exhibited an ethanol productivity of 0.78 g/(L·h), a sugar-to-ethanol conversion rate of 0.38 g/g, and a fermentation efficiency of 82.61%. Using the wild-type strain 1960 and a low-yielding strain 12.1 as controls, comparative genomics and transcriptomics were employed to elucidate the mechanism underlying the high ethanol production. Our findings are as follows: at the genomic level, there were 271 genomic structural variations. At the transcriptomic level, most genes involved in secondary metabolite synthesis, antibiotic synthesis, ribosomal pathways, amino acid biosynthesis, and oxidative phosphorylation pathways were down-regulated. Additionally, two key genes—*XYL1* (xylose reductase gene) and *XUT4* (high-affinity xylose transporter gene)—were significantly up-regulated. Through comprehensive integration of phenotypic comparison (e.g., fermentation performance of the high-yield strain 31.1 in yeast propagation and ethanol fermentation), comparative genomics, transcriptomics, and bioinformatics analyses of pathways involved in oxidative phosphorylation and the cell cycle (related to yeast cell growth), we identified 60 candidate key genes associated with high xylose-to-ethanol yield in *S. stipitis*. These genes are predominantly involved in the cell cycle pathway, including *CDC15* and *PHO81*. In conclusion, our study preliminarily reveals the mechanisms underlying the high xylose ethanol production of the high-yield strain at the genomic and transcriptomic levels.

## 1. Introduction

Bioethanol has gained public support for its ability to address issues such as fluctuations in crude oil prices, climate change, and the depletion of non-renewable fuel reserves. It is favored as a fuel blend component or alternative to fossil fuels, as it supports sustainable development and helps reduce reliance on fossil fuels. As a sustainable feedstock, renewable lignocellulosic biomass has demonstrated potential for replacing fossil fuels [1,2,3]. Lignocellulose-based biofuels are referred to as second-generation biofuels [1]. Second-generation bioethanol is a critical component of industrial biotechnology and a key approach for producing sustainable energy using lignocellulose. Notably, the efficient fermentation of hydrolyzed sugars derived from lignocellulose is crucial for improving ethanol titer and yield and reducing production costs [4,5].

Renewable feedstocks for bioethanol production mainly include sugars, starches, lignocellulosic biomass, and algae. Among these, lignocellulosic resources represent the most abundant renewable raw materials on Earth [6]. Sugarcane bagasse is a typical lignocellulosic resource, offering advantages such as concentrated availability (easy to utilize) and relatively low impurity content. China produces approximately 10 million tons of sugarcane bagasse annually, with the following composition: cellulose 32–48%, hemicellulose 19–24%, lignin 23–32%, and ash content around 4% [7]. However, due to technical limitations in bagasse conversion and utilization, most of it is directly burned as fuel, except for a small portion used in papermaking. This results in low overall biomass utilization efficiency and causes environmental pollution. Xylose is the second most abundant component in bagasse hydrolyzate. For this reason, the ability to produce ethanol via xylose fermentation is one of the key factors for the comprehensive utilization of bagasse fiber at present. However, wild-type Saccharomyces cerevisiae cannot utilize xylose for ethanol fermentation, and this has become one of the major bottlenecks in the industrial-scale production of cellulosic ethanol. Currently, microorganisms capable of utilizing xylose for ethanol production mainly include the following categories: (1) Yeasts: These include *Pachysolen tannophilus* [8], *Candida shehatae*, *S. stipitis*, *Pichia segobiensis* [9], *Brettanomyces* spp., and *Candida tenuis*; and (2) bacteria: These include *Thermoanaerobacter ethanolicus* (thermophilic anaerobic ethanologenic bacteria) and others. In addition, certain fungi—such as *Paecilomyces* sp. NF1 [10]—can also utilize xylose for ethanol production. Recently, two newly discovered yeast strains in Brazil, *Wickerhamomyces* sp. UFFS-CE-3.1.2 [11] and Spathaspora hagerdaliae UFMG-CM-Y303 [12], have also been shown to possess this capability. Statistics indicate that over 100 species of microorganisms capable of xylose utilization have been identified to date, with most of them being able to produce ethanol. Among these, *Candida shehatae*, *Pachysolen tannophilus*, and *S. stipitis* have been the most extensively studied [13,14]. For xylose ethanol fermentation, *S. stipitis* is regarded as one of the most ideal microorganisms currently available. This is primarily due to its ability to ferment various sugars in saccharified lignocellulose, including glucose, xylose, mannose, galactose, and cellobiose [5,15,16].

Recent engineering achievements include: thermotolerant S. cerevisiae producing 80.2 g/L ethanol via a two-stage temperature strategy [17]; rumen-derived xylose isomerase enabling 90% theoretical ethanol yield [18]; and engineered industrial *S. cerevisiae* strain ABX0928-0630 achieving near 100% xylose utilization efficiency with an ethanol yield of 0.48 g/g and a volumetric xylose consumption rate of 0.87 g/L·h [19]. These engineered yeasts, together with natural xylose-fermenting isolates, significantly expand the microbial toolkit for lignocellulosic biorefineries.

In 2007, Jeffries et al. [20] first completed the genome sequencing of *S. stipitis* (type strain CBS 6054). This strain has eight chromosomes with a genome size of 15.4 Mb, and its genome sequence has been deposited in the NCBI database. As of 6 June 2026, a total of four strains of *S. stipitis* with completed genome sequences have been deposited in the NCBI database, including the type strain CBS 6054, NRRL Y-7124, NRRL Y-50859, and Y-50861. In addition, Jeffries et al. [21] also performed comparative transcriptome sequencing of *S. stipitis* under two culture conditions: aerobic and microaerobic, with 50 g/L xylose as the carbon source at 30 °C and pH 5. They identified a total of 88 genes directly or indirectly associated with the pentose phosphate pathway (PPP), glycolysis, and tricarboxylic acid (TCA) cycle, including the three key genes *XYL1*, *XYL2*, and *XYL3*.

Zhu et al. [22] used transcriptomic and metabolomic analyses to study *S. stipitis* responses to three inhibitors (vanillin, HMF, and acetic acid). They found that sugar metabolism genes were significantly down-regulated, while amino acid metabolism (especially glutamine and asparagine synthesis) was induced to cope with stress. Shi et al. [23] characterized the physiological and metabolic traits of *S. stipitis* based on its genome sequence. Tian et al. [24] obtained a furfural-tolerant strain Y7-1 and, using qPCR and comparative proteomics, revealed differential expression of genes in the PPP, glycolysis, and TCA cycle, thereby elucidating the tolerance mechanism. Hilliard et al. [25] refined the redox balance and metabolic flux in *S. stipitis* by analyzing genome-scale metabolic network models and transcriptomic data.

Although *S. stipitis* exhibits potential in the fermentation of glucose and xylose from lignocellulosic biomass hydrolyzate for ethanol production, its ethanol yield and productivity—especially for xylose—have not met industrial requirements. Therefore, there is a need to construct more efficient strains [26].

Strain mutagenesis and improvement are traditional approaches, encompassing physical mutagenesis, chemical mutagenesis, and combined physical–chemical mutagenesis. Common physical mutagens include ultraviolet (UV) radiation and radioactive isotopes such as ^60^Co; typical chemical mutagens include diethyl sulfate (DES) and nitrosoguanidine (NTG). Ren Jia et al. [27] used ^60^Co to mutagenize Pachysolen tannophilus, while Zhao Lei et al. [28,29,30] employed UV and microwave mutagenesis on Pachysolen tannophilus. In all cases, the xylose-to-ethanol fermentation performance of the resulting mutants was improved. Watanabe et al. [31] performed UV mutagenesis on *S. stipitis* strain NBRC1687 to screen and select mutants, obtaining an excellent mutant strain PXF-58. When fermenting 114 g/L xylose, the ethanol concentration of PXF-58 reached 5.45%—38.68% higher than that of the original strain (3.93%).

However, current mutagenesis studies on *S. stipitis* have mostly focused on phenotypic screening, and few have integrated comparative genomics with transcriptomics to dissect the mechanisms underlying high-yield phenotypes. Comparative genomics and transcriptomics were prioritized over metabolomics or proteomics because mutagenesis and long-term domestication primarily induce genetic variations and transcriptional reprogramming; these are more directly captured at the DNA and RNA levels for identifying causal mutations and key regulatory genes underlying the high-yield phenotype.

Based on this, in the present study, the high-ethanol-yielding strain 31.1 and the low-yielding strain 12.1 were obtained from *S. stipitis* 1960 through Co^60^ mutagenesis combined with long-term xylose domestication. Using the original strain, the high-yielding strain, and the low-yielding strain as comparative objects, we systematically analyzed genomic structural variations and differential gene expression by comparative genomics and transcriptomics. The objectives of this study were: (1) to reveal the molecular mechanism underlying the high xylose-to-ethanol yield in strain 31.1; (2) to identify candidate key genes associated with efficient xylose conversion and high ethanol production; and (3) to provide a theoretical basis for future metabolic engineering of *S. stipitis* or other industrial strains.

## 2. Materials and Methods

(1) Strains

*S. stipitis* strains: The wild-type strain CICC1960 (purchased from the China Center of Industrial Culture Collection, CICC; Beijin, China), abbreviated as strain 1960, is preserved in our laboratory at −80 °C in glycerol with a final concentration of 25% (*v*/*v*) under ultralow-temperature conditions.

(2) Culture Media (all in *w*/*v*, %)

① YPX: 2% xylose, 2% peptone, and 1% yeast extract; sterilized at 121 °C for 20 min, natural pH; 2% agar was added for solid culture media.

② YPX4: 4% xylose, 2% peptone, and 1% yeast extract; sterilized at 121 °C for 20 min, natural pH.

③ Culture media for screening yeasts capable of growing in non-detoxified sugarcane bagasse hydrolysate: Acid-treated non-detoxified sugarcane bagasse hydrolysate, supplemented with 2% peptone and 1% yeast extract; pH was adjusted to 3.8–4.0 with sulfuric acid. Culture media for ethanol production via fermentation of non-detoxified sugarcane bagasse hydrolysate: Concentrated non-detoxified sugarcane bagasse hydrolysate, supplemented with 15% glucose, 2% peptone, and 1% yeast extract; pH was adjusted to 3.8–4.0 with sulfuric acid. A total of 30% glucose was added as feeding material.

The peptone and yeast extract were purchased from OXOID (Thermo Fisher Scientific, Basingstoke, UK); xylose was from Sigma-Aldrich (Merck KGaA, Darmstadt, Germany); other chemicals were from Sinopharm Chemical Reagent Co., Ltd. (Shanghai, China).

(3) Determination of Ethanol Content

We referred to the method described in Lu et al. [32]. Ethanol content was determined by gas chromatography. The brand and model of the gas chromatograph are Agilent and 6890N/7890A.

The internal standard used was 10% acetonitrile (*v*/*v*). For sample pretreatment, after centrifugation of the sample (for molasses, additional treatment with basic lead acetate was required), 600 μL of the supernatant was aspirated, mixed with an equal volume of 10% acetonitrile, then 600 μL of deionized water was added to make up the volume. The mixture was homogenized for ethanol content determination. A 1 μL microsyringe was used for injection, with an injection volume of 0.1 μL. The ethanol content (% (*v*/*v*)) was calculated based on the peak areas of ethanol and acetonitrile, using the following formula: Ethanol content (% (*v*/*v*)) = (Peak area of ethanol/Peak area of acetonitrile) × 0.9724 (Note: 0.9724 is a constant, derived from the linear regression equation of the standard curve. The standard curve was established by measuring the peak areas of ethanol and acetonitrile under the same conditions using the internal standard method).

Gas Chromatography Conditions

Detector: Flame Ionization Detector (FID); detector temperature: 325 °C. Gas flow rates: H_2_ (hydrogen) = 40 mL/min; air = 450 mL/min; make-up gas (N_2_, nitrogen) = 28.7 mL/min. Injection port: Pressure = 16.91 Psi; injector heater temperature = 250 °C; carrier gas = N_2_ (nitrogen); split ratio = 50:1; and total N_2_ flow rate = 70.5 mL/min. Column: Model Zebron ZB-WAX; column flow rate = 1.3 mL/min; and column temperature = 100 °C.

(4) ^60^Co Mutagenesis and Screening

The activated *S. stipitis* strain 1960 was inoculated into YPX medium and cultured at 30 °C with shaking at 180 rpm until the logarithmic phase. The cells were harvested by centrifugation (12,000 rpm, 5 min), washed three times with sterile physiological saline, and resuspended in physiological saline to a final concentration of 1 × 10^8^ cells/mL. Nine different radiation doses (0.4, 0.6, 0.8, 1.0, 1.2, 1.4, 1.6, 1.8, and 2.0 kGy) were applied for mutagenesis using a ^60^Co radiation source at an approved irradiation center.

Strain screening: The mutagenized cell suspension was diluted with physiological saline to 1 × 10^5^ cells/mL and spread onto YPX agar plates. Approximately 10 plates were used for each dose. After incubation at 30 °C for 3 days, single colonies were picked and inoculated into 15% FM medium for fermentation. Fermentation was carried out at 30 °C with shaking at 130 rpm for 60–72 h. Samples were taken at 48 h, 60 h, 72 h, and 80 h to measure the ethanol content in the fermentation broth. Strains with higher ethanol production were selected as primary candidates.

Strain purification: The selected yeast strains were streaked onto YPD agar plates and purified three times consecutively. The purified strains were stored in 25% (*v*/*v*) glycerol at −80 °C.

(5) Strain domestication

The xylose-to-ethanol high-yielding strains obtained through ^60^Co mutagenesis and screening were separately inoculated into 10 °Bx and 20 °Bx sugarcane molasses (both autoclaved prior to use). The cultures were incubated at 30 °C with shaking at 180 rpm for more than three months for domestication. Subsequently, the strains were re-screened, purified, and preserved following the same procedures as described above for the screening and purification of ^60^Co-mutagenized strains.

(6) Xylose Ethanol Fermentation

Xylose-ethanol fermentation was performed as described previously [33]. A total of 15% FM fermentation medium or concentrated sugarcane bagasse hydrolysate was used as the fermentation substrate. After three rounds of strain activation, the seed culture was inoculated into 15% FM fermentation medium or concentrated sugarcane bagasse hydrolysate at a 10% inoculation amount. The fermentation was carried out in 250 mL Erlenmeyer flasks with a liquid volume of 50 mL, cultured at 30 °C with shaking at 130 rpm. Samples were taken every 8 h, and two biological replicates were set up for the experiment.

(7) Determination of Xylose Content

Determination of xylose content was performed as described previously [33], same as above. The determination was conducted using the DNS method, with a 4 mL reaction system, except that the OD520 value (optical density at 520 nm) was measured. Two parallel determinations were set up.

Comparative Genomics and Transcriptomics Study of *S. stipitis*

(8) Genomic DNA Extraction and Detection

High-yield strains, low-yield strains, and the wild-type *S. stipitis* strain were first activated in YPX medium. After activation, cells in the logarithmic growth (approximately 2 × 10^8^ cells/mL) phase were harvested for genomic DNA extraction.

Agarose gel electrophoresis was used to determine the purity and integrity of the sample DNA, while Qubit (a nucleic acid quantification instrument) was employed for DNA quantification. Three parallel experiments were set up for each strain.

(9) Genomic Sequencing and Analysis

This work was undertaken by Beijing Novogene Technology Co., Ltd. (Beijing, China), mainly including library construction, on-instrument sequencing, and analysis. After data processing, the sequences of the three strains were all subjected to draft genome assembly prior to analysis. The main analyses included: component analysis, functional annotation (all combined with de novo transcript assembly from transcriptome sequencing), comparative genomic analysis, etc. Three biological replicates were set up for each strain.

(10) Total RNA Extraction

Total RNA extraction was performed as described previously [34]. Ethanol fermentation was conducted using 15% (*w*/*v*) xylose, and cells were harvested at 12 h and 60 h of fermentation (the time point when ethanol content reached its maximum). Each aliquot of cells was archived: after snap-freezing in liquid nitrogen, they were stored at −80 °C. Total RNA was extracted from the cells using the TRIzol method [35]. The purity of RNA was then detected using a Nano Photometer spectrophotometer, including the OD260/280 and OD260/230 ratios; RNA integrity was accurately assessed using an Agilent 2100 bioanalyzer. Three biological replicates were performed.

(11) Transcriptome Sequencing and Analysis

This work was undertaken by Beijing Novogene Technology Co., Ltd., mainly including data quality control, reference genome alignment, quantitative analysis, differential analysis, and enrichment analysis, among others. In addition, de novo transcript assembly was performed for the above three *S. stipitis* strains: for the three strains at two fermentation time points, from the three biological replicates, the two with the highest correlation (i.e., Pearson correlation coefficient R^2^) were selected for assembly, resulting in a total of 12 assemblies.

(12) qPCR Validation [36]

From the transcriptome sequencing results, we selected some significantly differentially expressed up-regulated and down-regulated genes (including several key genes) and designed primers for them. After reverse transcription of total RNA, quantitative real-time PCR (qRT-PCR) amplification was performed.

For each RNA sample, three technical replicates were set up. Data analysis was conducted using the 2^−ΔΔCt^ method [36].

(13) Data statistical analysis

The experimental data were statistically analyzed using Microsoft Excel (Office 2019, Microsoft, Redmond, WA, USA) and SPSS (version 17.0, IBM, Armonk, NY, USA). Comparisons between two independent groups were performed using Student’s *t*-test. Statistical significance was set at *p* < 0.05. Asterisks indicate statistical significance (* *p* < 0.05, ** *p* < 0.01, *** *p* < 0.001), while ns denotes no significant difference (*p* ≥ 0.05).

(14) Sequencing and bioinformatics analysis

Total RNA was extracted from each sample, and RNA integrity and concentration were assessed using a Qubit fluorometer and a bioanalyzer. For transcriptomic sequencing, mRNA was enriched from total RNA using Oligo(dT) magnetic beads. After fragmentation, first-strand cDNA was synthesized using random hexamer primers, followed by second-strand cDNA synthesis with dUTP instead of dTTP to preserve strand orientation. After end repair, A-tailing, adapter ligation, fragment selection, amplification, and purification, the strand-specific libraries were prepared. Library quality and quantity were verified by Qubit, real-time PCR, and bioanalyzer for fragment size distribution.

For whole-genome sequencing (WGS), genomic DNA was fragmented, and libraries were prepared following the standard protocol with end repair, A-tailing, adapter ligation, fragment selection, amplification, and purification. Library quality and quantity were verified by Qubit, real-time PCR, and bioanalyzer.

All libraries were sequenced on an Illumina platform with paired-end 150 bp (PE150) reads. For RNA-Seq, approximately 6 Gb of clean data were generated per sample.

Raw reads were first processed by fastq software (FASTQ_Sanger or Illumina +1.8) to remove adapter-containing reads, reads with excessive ambiguous bases (N content > 10%), and low-quality reads (Q ≤ 5 in >50% of bases), yielding clean reads. Q20, Q30, and GC content were calculated for the clean data.

Clean reads were aligned to the reference genome using HISAT2, which builds an index of the reference genome and performs alignment based on the gene annotation file. Gene expression levels were quantified by featureCounts and normalized as FPKM (fragments per kilobase of transcript per million mapped reads). Differential expression analysis between groups was performed using the DESeq2 R package, with the Benjamini–Hochberg method applied to control the false discovery rate. The significance threshold was set at padj < 0.05 and |log_2_FoldChange| > 0.

GO enrichment analysis of differentially expressed genes was performed using clusterProfiler, correcting for gene length bias. GO terms with a corrected *p*-value < 0.05 were considered significantly enriched. KEGG pathway enrichment analysis was also performed using clusterProfiler.

Gene set enrichment analysis (GSEA) was performed using the local version 4.3.2 of GSEA software (http://www.broadinstitute.org/gsea/index.jsp; accessed on 6 May 2025) against the GO and KEGG datasets of the species.

Protein–protein interaction (PPI) analysis of differentially expressed genes was based on the STRING database. For species present in the database, the network was constructed by extracting the target gene list from the database; otherwise, diamond was used to align the target gene sequences against selected reference protein sequences, and the network was established based on known interactions of the selected reference species.

Variant calling (SNPs and InDels) was performed using GATK.

Weighted gene co-expression network analysis (WGCNA) was performed using the WGCNA R package to identify gene sets with highly coordinated changes and to associate gene modules with phenotypic traits.

## 3. Results

### 3.1. Study on Mutagenesis and Breeding of S. stipitis

For *S. stipitis*, since Watanabe et al. have already conducted mutagenesis and screening using ultraviolet (UV) radiation, UV radiation was not considered for mutagenesis in this study. Ren Jia et al. used ^60^Co to mutagenize Pachysolen tannophilus and achieved good mutagenesis effects. In addition, a radiation center could conduct ^60^Co irradiation, and the operation of ^60^Co mutagenesis on *S. stipitis* is relatively simple. Considering these factors comprehensively, this study carried out ^60^Co mutagenesis on *S. stipitis*, and the results are as follows.

The wild-type strain 1960 of *S. stipitis* was mutagenized using 9 different ^60^Co doses, followed by plating and inoculation on YPX plate medium. It was found that when the dose increased to 1.2 kGy, the number of colonies growing on the plates decreased, and the number of colonies showed a decreasing trend with the gradual increase in dose. Single colonies on the plates were randomly selected (10 strains were selected for each dose, totaling 90 strains). All strains were purified by streaking on plates three times before being preserved.

The 90 strains were subjected to xylose ethanol fermentation (in batches), and samples were taken to determine ethanol content. Using the ethanol content of the wild-type strain as a control, the effect of mutagenesis was compared during primary screening (see Figure S1). The primary screening results of the strains showed that more positive mutant strains were obtained at low doses (0.4–1 kGy); especially at the dose of 1 kGy, among the 10 selected strains, only 1 was a negative mutant strain. When the dose reached 1.2 kGy, most of the obtained mutants were negative mutant strains, and the xylose ethanol yield of the positive mutant strains was not significantly higher than that of the wild-type strain. The results at doses of 1.4–2 kGy were similar to those at 1.2 kGy.

These results indicate that low doses of ^60^Co are relatively ideal for mutagenizing *S. stipitis*, as they yield more positive mutant strains. The strains were then subjected to secondary screening, and the results are shown in Table S1. It can be seen that strain 1K-9 was the most excellent one.

On the basis of obtaining several excellent strains through the aforementioned mutagenesis and screening, these strains were respectively inoculated into 10 °Bx and 20 °Bx sugarcane molasses media, then cultured and acclimated at 30 °C with shaking at 180 rpm for more than 3 months. From nearly 50 candidate strains, multiple strains capable of rapidly utilizing xylose for ethanol production were selected. These strains were subjected to xylose ethanol fermentation, and ethanol content was determined to finally screen out the superior strain 31.1.

When strain 31.1 fermented with 15% (*w*/*v*) xylose for 48 h, its ethanol content exceeded 5.40% (*v*/*v*), which was 35% higher than that of the wild-type strain 1960 (4.00%, *v*/*v*) and higher than that of strain 1K-9 (4.26%, *v*/*v*). Based on these fermentation characteristics, three typical strains were selected for this study: the high-yield strain 31.1, the wild-type strain 1960, and the low-yield strain 12.1.

The three strains were each fermented with 15% (*w*/*v*) xylose, and their ethanol yields at 60 h are shown in Figure S2a. In contrast to the 35% improvement observed at 48 h, the ethanol yield of strain 31.1 increased by 14.13% compared with strain 1960 at 60 h (highly significant, *p* < 0.001); the ethanol yield of the low-yield strain 12.1 decreased by 25.61% compared with the wild-type strain (highly significant, *p* < 0.001); and the ethanol yield of strain 31.1 was 53.42% higher than that of the low-yield strain (highly significant, *p* < 0.001).

The three strains were cultured at 30 °C with shaking at 200 rpm for 60 h, and their relative respiratory intensities are shown in Figure S2b. The respiratory intensities of both the high-yield strain 31.1 and the low-yield strain 12.1 were significantly lower than that of the original strain (*p* < 0.01). Specifically, the respiratory activity of the high-yield strain 31.1 was 59% of that of the original strain, while that of the low-yield strain 12.1 was 49.85% of that of the original strain.

A 20 μL seed culture was inoculated into 6 mL of YPX medium, followed by static culture at 30 °C for 48 h and 60 h. The differences in gas production among the three strains are shown in Figure S3. Gas production was quantified with the full gas filling of the inverted Durham tube defined as 100%. The results showed that the wild-type strain had the fastest gas production: its gas production accounted for approximately 1/3 of the Durham tube volume at 48 h and filled the tube at 60 h. This was followed by the high-yield strain 31.1, whose gas production accounted for approximately 1/5 of the tube volume at 48 h and filled the tube at 60 h—with no significant difference between the wild-type strain and strain 31.1 (*p* ≥ 0.05). The low-yield strain had the slowest gas production (significant, *p* < 0.05): its gas production accounted for approximately 1/10 and 2/5 of the tube volume at 48 h and 60 h, respectively, and it did not fill the tube until 67 h—7 h later than the other two strains.

**Figure 1:**
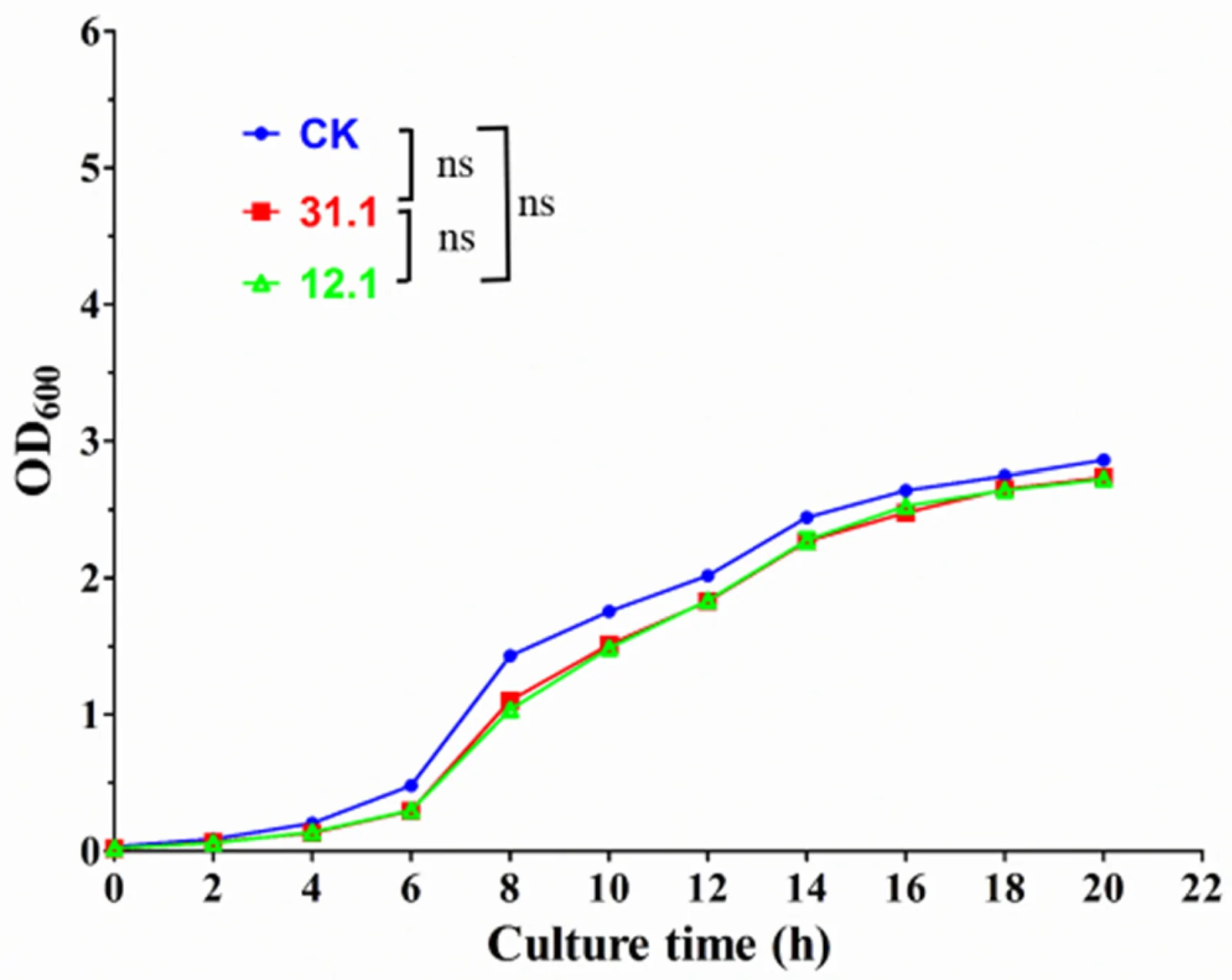
Growth curve. Note: Cultured condition: 30 °C, 200 rpm in YPX. Data are mean ± SD of *n* = 2 biological replicates. ns: not significant, *p* ≥ 0.05 (Student’s *t*-test). Differences among samples were not significant.

The three strains were inoculated into YPX medium and cultured at 30 °C with shaking at 180 rpm; their growth curves are shown in Figure 1. The results indicated that the wild-type strain had the fastest growth rate, while the two mutant strains exhibited similar growth rates—both slower than that of the wild-type strain. However, no significant differences were observed among the three strains (*p* ≥ 0.05).

In fact, the parental strain of the high-yield strain 31.1 is strain 1K-9, and the latter had a growth rate similar to that of the wild-type strain (with no significant difference). After strain 1K-9 was acclimated to sugarcane molasses, the selected strain 31.1 showed improved xylose ethanol fermentation performance, but its growth rate became slightly slower.

**Figure 2:**
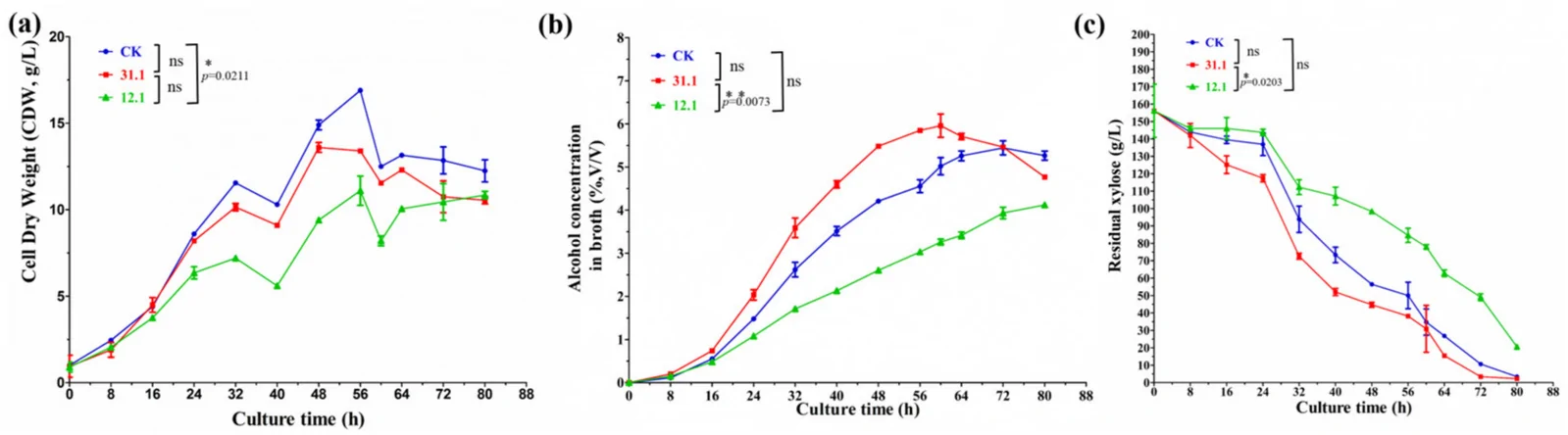
Fermentation curve. Note: Cultured condition: 30 °C, 130 rpm in 15% FM. (**a**) Biomass (cell dry weight) curve; (**b**) ethanol curve; (**c**) residual xylose curve. Data are mean ± SD of *n* = 2 biological replicates. Asterisks indicate significant differences (Student’s two-tailed *t*-test): * *p* < 0.05, ** *p* < 0.01. ns: not significant (*p* ≥ 0.05).

In addition, the three strains were inoculated into 15% FM medium for fermentation at 30 °C with shaking at 130 rpm; their fermentation curves are shown in Figure 2.

The results showed that when 15% xylose (*w*/*v*) was used as the substrate, the following occurred.

Growth rate: The wild-type strain still had the fastest growth rate, followed by the high-yield strain 31.1 (Figure 2a), with no significant difference between the two; the low-yield strain had the slowest growth rate, which was slightly slower than that of the high-yield strain (no significant difference between them), but showed a significant difference compared with the wild-type strain (*p* < 0.05).

Ethanol production: The high-yield strain could rapidly utilize xylose for ethanol production and achieved the highest ethanol yield (Figure 2b), followed by the wild-type strain (no significant difference between the two); the low-yield strain had the lowest ethanol yield, and there was an extremely significant difference in ethanol content between the low-yield strain and the high-yield strain (*p* < 0.01).

Key fermentation parameters: The xylose ethanol productivities of the high-yield, wild-type, and low-yield strains were 0.78 g/(L·h), 0.66 g/(L·h), and 0.43 g/(L·h), respectively; their corresponding xylose-to-ethanol conversion rates were 0.3755 g/g, 0.3264 g/g, and 0.2887 g/g; and their fermentation efficiencies were 82.61%, 71.74%, and 63.04%, respectively.

Residual xylose and utilization rate: The high-yield strain 31.1 had the lowest residual xylose content, followed by the wild-type strain (no significant difference between them); the low-yield strain had the highest residual xylose content, which showed a significant difference compared with the high-yield strain but no significant difference compared with the wild-type strain (Figure 2c). After 80 h of fermentation, the xylose utilization rates of the high-yield, wild-type, and low-yield strains were 98.52%, 97.81%, and 86.83%, respectively.

The above results indicate that the xylose ethanol fermentation performance of the high-yield strain 31.1 is superior to that of the wild-type strain and the low-yield strain.

### 3.2. Comparison of the Fermentation Performance Between the 31.1 Strain and Other Strains

The high-yield strain 31.1 obtained in this study exhibited a sugar-to-ethanol conversion rate of 0.38 g/g, a fermentation efficiency of 82.61%, and an ethanol productivity of 0.78 g/(L·h) when fermenting 15% (*w*/*v*) xylose. The corresponding values for the wild-type strain 1960 were 0.33 g/g, 71.74%, and 0.66 g/(L·h), while those for the low-yield strain 12.1 were 0.29 g/g, 63.04%, and 0.43 g/(L·h) (Table 1).

Among the xylose-to-ethanol-producing strains reported to date, the fermentation performances of selected strains are as follows. When fermenting 150 g/L and 200 g/L xylose, *Paecilomyces* sp. NF1 [10] achieved ethanol concentrations of 7.56% and 9.31%, sugar-to-ethanol conversion rates of 0.39 g/g and 0.38 g/g, and a fermentation efficiency of 75% in both cases. However, the fermentation period was as long as 12–13 days, with an ethanol productivity of only 0.21–0.24 g/(L·h). *Spathaspora passalidarum* [37] achieved the highest ethanol yield of 0.44 g/g and an ethanol productivity of 1.02 g/(L·h) under conditions of 100 g/L xylose at 32 °C. *Zymomonas mobilis* [38] A3 reached a xylose fermentation efficiency of 88.20%. *S. stipitis* CBS 5576 [39] exhibited a fermentation efficiency of 97.83%, but its ethanol productivity was only 0.34 g/(L·h). *S. stipitis* UFMG-CM-Y2303 and UFMG-CM-Y2108 [40] exhibited fermentation efficiencies of 82.00% and 83.00%, respectively, but had ethanol productivities of only 0.39 g/(L·h) and 0.40 g/(L·h), respectively. The *S. stipitis* strain PXF58 produced an ethanol concentration of 5.45% from 114 g/L xylose, with a sugar-to-ethanol conversion rate of 0.38 g/g, a fermentation efficiency of 82.00%, and an ethanol productivity of 0.60 g/(L·h) [31]. The *Pachysolen tannophilus* strain 800-3, when fermented with 50 g/L xylose for 72 h, showed a sugar-to-ethanol conversion rate of only 0.14 g/g, a fermentation efficiency of 30.43%, and an ethanol productivity of 0.08 g/(L·h) [27].

Therefore, the xylose-to-ethanol fermentation performance of the high-yield strain 31.1 ranks among the advanced levels in domestic studies of the same type.

**Table 1:** Comparison of xylose ethanol fermentation performance.

Name of Strain	Xylose/(g/L)	Conversation of Xylose to Ethanol/(g/g)	Fermentation Efficiency/%	Ethanol Yield/[g/(L·h)]	Ref.
*Paecilomyces* sp. NF1	150	0.39	75.00	0.21	[10]
*Paecilomyces* sp. NF1	200	0.38	75.00	0.24	[10]
*Pachysolen tannophilus* RL-171	160	0.10	21.74	-	[41]
*Pachysolen tannophilus* RL-171	200	0.06	13.04	-	[41]
*Pachysolen tannophilus* NRRL-Y2460	115	0.24	52.17	-	[42]
*Pachysolen tannophilus* NRRL-Y2460	158	0.21	45.65	-	[42]
*Candida shehatae* CBIR-Y492	50	0.36	78.26	-	[43]
*Candida shehatae* CBIR-Y492	90	0.29	63.04	-	[43]
*Candida tropicalis* ATCC 1369	75	0.11	23.91	-	[44]
*S. stipitis* CBS 5576	50	0.45	97.83	0.34	[45]
*Pichia segobiensis* CBS 6857	20	0.25	54.35	0.02	[46]
*S. stipitis* NRRL Y-7124	30	0.28	60.87	-	[39]
*Spathaspora passalidarum* NRRL Y-27907	30	0.35	76.09	-	[37]
*Pestalotiopsis* sp. XE-1	97.71	0.34	73.91	0.06	[47]
*Zymomonas mobilis* A3	100	0.45	88.20	0.94	[38]
*Spathaspora hagerdaliae* UFMG-CM-Y303	28.2	0.28–0.36	60.87–78.26	0.06–0.13	[48]
*Candida maltosa* UFMG-CM-Y2131	30	0.12	23.97	0.09	[40]
*Scheffersomyces* sp. UFMG-CM-Y365	30	0.25	49.81	0.22	[40]
*S. stipitis* UFMG-CM-Y2303	30	0.42	82.00	0.39	[40]
*S. stipitis* UFMG-CM-Y2108	30	0.42	83.00	0.40	[40]
*Spathaspora boniae* UFMG-CM-Y306	30	0.19	38.02	0.25	[40]
*Spathaspora boniae* UFMG-CM-Y363	30	0.22	43.87	0.29	[40]
*Sugiyamaella xylolytica* UFMG-CM-Y348	30	0.14	28.00	0.08	[40]
*Wickerhamomyces* sp. 1 UFMG-CM-Y6241	30	0.14	28.54	0.05	[40]
*Saccharomyces cerevisiae* CAT-1-XIT(pRS42K::XI)	30	0.31	67.39	0.10	[49]
*Pachysolen tannophilus* 800-3	50	0.14	30.43	0.08	[27]
*Pachysolen tannophilus* 1770-7	20	0.17	36.96	-	[29]
*Pachysolen tannophilus* 1770-11	20	0.18	39.13	-	[29]
*S. stipitis* NBRC 1678	114	0.27	59.12	0.50	[31]
*S. stipitis* PXF58	114	0.38	82.00	0.60	[31]
*S. stipitis* CICC1960 ^a^	150	0.31	67.39	0.55	This study
*S. stipitis* 1K-9 ^a^	150	0.34	73.91	0.61	This study
*S. stipitis* 12.1 ^b^	150	0.29	63.04	0.43	This study
*S. stipitis* CICC1960 ^b^	150	0.33	71.74	0.66	This study
*S. stipitis* 31.1 ^b^	150	0.38	82.61	0.78	This study

Note: ^a^: Average value at 84 h of fermentation; ^b^: Average value at 60 h of fermentation.

### 3.3. Mining of Key Genes for High-Yield Xylose Ethanol in S. stipitis

#### 3.3.1. Comparative Genomics Study of Three *S. stipitis* Strains

Based on the previous screening and construction of the high-yield strain 31.1 and low-yield strain 12.1, a comparative genomics study was conducted on the high-yield, low-yield, and wild-type strains.

The wild-type strain 1960, high-yield strain 31.1, and low-yield strain 12.1 were inoculated into YPX medium and cultured to the logarithmic growth phase. Three biological replicates were set up for each of the three strains, resulting in a total of nine samples. Genomic DNA was extracted from each sample, and the results of agarose gel electrophoresis detection are shown in Figure S4. The quantitative detection results of genomic DNA are presented in Table S2; the results indicated that the genomic DNA of the nine samples was of high quality, meeting the requirements for library construction and sequencing. The results of library construction are shown in Figures S5–S13, which demonstrated that the library quality was ideal and suitable for on-instrument sequencing.

The raw data were processed through filtering, adapter sequence removal, and other steps, and the resulting clean data are shown in Table 2. The results revealed that the Q30 values of all nine samples were above 92%. In addition, the comparison results of coverage with the reference genome are shown in Table 3; it can be seen that the proportion of reads mapped to the reference genome exceeded 93% for all samples. These results confirm that the sequencing data are reliable and suitable for subsequent analysis.

Draft genome assembly was performed on the aforementioned nine datasets, yielding three sets of data corresponding to the three strains; the results are shown in Table S3. The proportion of Q30 bases in all three sets exceeded 92.6%. In addition, the statistical results of coverage and sequencing depth are presented in Table S4, and the contig statistics are shown in Table S5. These results confirm the reliability of the data.

Furthermore, to improve the accuracy of analyses on genome composition, gene count, and other aspects, de novo transcript assembly was conducted for the three strains based on the subsequent research content (*S. stipitis* transcriptome sequencing results). For the three strains at two fermentation time points: from the three biological replicates of each strain–time point combination, the two with the highest correlation were selected for assembly, resulting in a total of 12 transcript assemblies. Genomic composition and other characteristics were analyzed by referencing these transcripts, with the results shown in Figure S14.

The results indicated that there were certain differences in genome composition among the three strains: the number of predicted genes (>2500 bp) in the wild-type strain 1960 was 676, which was fewer than that in strain 31.1 (705) and strain 12.1 (706). Overall, strain 1960 had the fewest predicted genes, followed by strain 31.1, and strain 12.1 had the most (Table S6). It should be noted that the difference between the high-yield strain 31.1 and the wild-type strain 1960 was approximately 4.3%. For draft genome assemblies based on Illumina short-read sequencing, this level of variation falls within the typical range of technical variation, as previous studies have shown that short-read assemblies can miss substantial pangenomic gene variation [50]. Such variation may be influenced by assembly fragmentation, repeat region handling, or gene prediction parameters. Therefore, the biological significance of this difference requires further validation (e.g., by long-read sequencing or qPCR copy-number analysis). The analysis results of non-coding RNA (ncRNA) and repetitive sequences are shown in Table S7 and Table S8, respectively.

By comparing different databases, the genomic functions of the three strains were analyzed and compared, with the results shown in Table S9. The results indicated that across 13 database categories, the high-yield strain 31.1 had higher counts in all categories than the wild-type strain, but slightly lower counts than the low-yield strain (with counts equal to those of the low-yield strain in two database categories). The results of Gene Ontology (GO) analysis are shown in Figures S15–S17, which revealed that the three strains exhibited certain differences in their GO analysis outcomes. The results of Kyoto Encyclopedia of Genes and Genomes (KEGG) analysis are presented in Figures S18–S20, indicating that there were also certain differences among the three strains in this regard. The results of CAZy (Carbohydrate-Active enZYmes) database analysis related to carbohydrate enzymes are shown in Figure S21 and Table S10. The results demonstrated that: The high-yield strain 31.1 had the largest number of glycosyltransferase genes (81), while the wild-type strain and low-yield strain had slightly fewer, with 78 each; the low-yield strain had the largest number of glycoside hydrolase genes (70); and the high-yield strain and wild-type strain had slightly fewer, with 62 and 63, respectively. These results indicated that there were certain differences in genomic functions among the three strains. The increased number of glycosyltransferase genes in the high-yield strain 31.1 may contribute to enhanced cell wall remodeling and protein glycosylation, which are critical for tolerance to ethanol and osmotic stress during xylose fermentation. Studies have shown that ethanol stress activates the cell wall integrity (CWI) pathway in yeast, inducing the expression of cell wall-remodeling genes including CRH1 (encoding a chitin transglycosylase, a glycosyltransferase) [51]. Furthermore, proper protein glycosylation is essential for the activity and stability of secreted enzymes and for the functionality of cell wall stress sensors [52,53]. The increased glycosyltransferase gene repertoire in strain 31.1 may therefore support superior fermentation performance through enhanced cell wall integrity and optimized glycosylation of xylose-metabolizing enzymes.

Comparative genomics analysis was performed using the high-yield strain 31.1 as the reference genome. The results of InDel (Insertion/Deletion) analysis are shown in Table S11, which indicated that the number of InDels in strain 12.1 (low-yield) was relatively high, while that in strain 1960 (wild-type) was significantly lower. In addition, the statistical results of InDel mutation counts are presented in Table S12. These results revealed that the number of InDels in the coding sequences (CDS) of strain 12.1 was also higher than that of strain 1960. The types of InDels are shown in Table S13 and Figure S22; it can be seen that there were slightly more differences between the low-yield strain and the high-yield strain than between the wild-type strain and the high-yield strain. The analysis results of genomic structural variations (SV) are shown in Figure S23 (for strain 1960) and Figure S24 (for strain 12.1): compared with the wild-type strain, the high-yield strain had a total of 271 genomic structural variations; in contrast, compared with the low-yield strain, the high-yield strain had a total of 223 genomic structural variations. By comparing the synteny between different genomes, information such as insertions and deletions was identified to analyze gene structural variation. The results are shown in Figure S25 (for strain 1960) and Figure S26 (for strain 12.1). The above results indicated that the differences between the high-yield strain and the wild-type strain, as well as between the high-yield strain and the low-yield strain, were relatively significant. Specifically, there were certain differences in the genomic structure of the high-yield strain 31.1 compared with both the wild-type strain and the low-yield strain.

#### 3.3.2. Comparative Transcriptomics Study of Three *S. stipitis* Strains

Based on the above analysis of the roles of genes in yeast growth and ethanol fermentation, comparative transcriptomics analysis was conducted on three strains—low-yield strain 12.1 (abbreviated as L, the same below), high-yield strain 31.1 (abbreviated as H, the same below), and wild-type strain 1960 (abbreviated as CK, the same below)—during xylose (15%, *w*/*v*) ethanol fermentation. Samples were collected at the early growth stage (12 h) and the stage of maximum ethanol content (60 h) for analysis.

Low-yield strain 12.1 (L), high-yield strain 31.1 (H), and wild-type strain 1960 (CK) were inoculated into 15% FM fermentation medium for xylose ethanol fermentation. Samples were collected at 12 h and 60 h, and total RNA was extracted from the samples (three biological replicates for each of the three strains at two time points, totaling 18 samples). The results of agarose gel electrophoresis are shown in Figure S27, and the quality test results are presented in Table S14. The total RNA of most of these samples met the conditions mentioned in Section 3.3.1, and all samples were graded A in the quality assessment. These results indicate that the total RNA of the samples was of extremely high quality, suitable for library construction.

The libraries of the aforementioned 18 total RNA samples were subjected to on-instrument sequencing, and the summary of data quality results is shown in Table S15. After filtering the raw data, the minimum number of clean reads was 41,392,204 (corresponding to the sample H_60h_1), and the minimum number of clean bases (after raw data filtering) was 6.21 Gb. The Q30 values of all data in this batch exceeded 93.9%. These results indicate that the overall quality of the sequencing data was relatively ideal and reliable.

Pearson correlation coefficient (R^2^) analysis among the three biological replicates of each of the six samples is shown in Figure S28, with detailed R^2^ values presented in Figures S29–S34. The results revealed that: Samples L_60_2 and L_60_3 showed good correlation, but their correlation with L_60_1 was less ideal; samples CK_60_1 and CK_60_3 showed good correlation, but their correlation with CK_60_2 was less ideal; and the three biological replicates of the other four samples showed good correlation. These results confirm that the biological replicates of the six samples were relatively ideal, and the data were reliable.

Principal component analysis (PCA) was performed on the gene expression values (FPKM) of all samples, with the results shown in Figure S35. The results indicated that the six sample groups were relatively scattered from each other, while samples within the same group clustered together—these results were relatively ideal.

The obtained transcriptome sequencing data were aligned with the *S. stipitis* genome sequence from the Ensembl Database. The mapping statistics are shown in Table S16. The results indicated that for all 18 samples, the proportion of reads mapped to the reference genome exceeded 94%, and among these, the proportion of reads mapped to unique positions on the reference genome sequence exceeded 93%. These results confirm that the data are reliable and suitable for subsequent analysis.

After calculating the expression values (FPKM) of all genes in each sample, this study compared the gene density among different samples (Figure S36). The distribution of gene expression levels across different samples was visualized using box plots (see Figure S37) and a Violin chart (see Figure S38). The results indicated that there were certain differences in gene expression levels among the samples.

**Figure 3:**
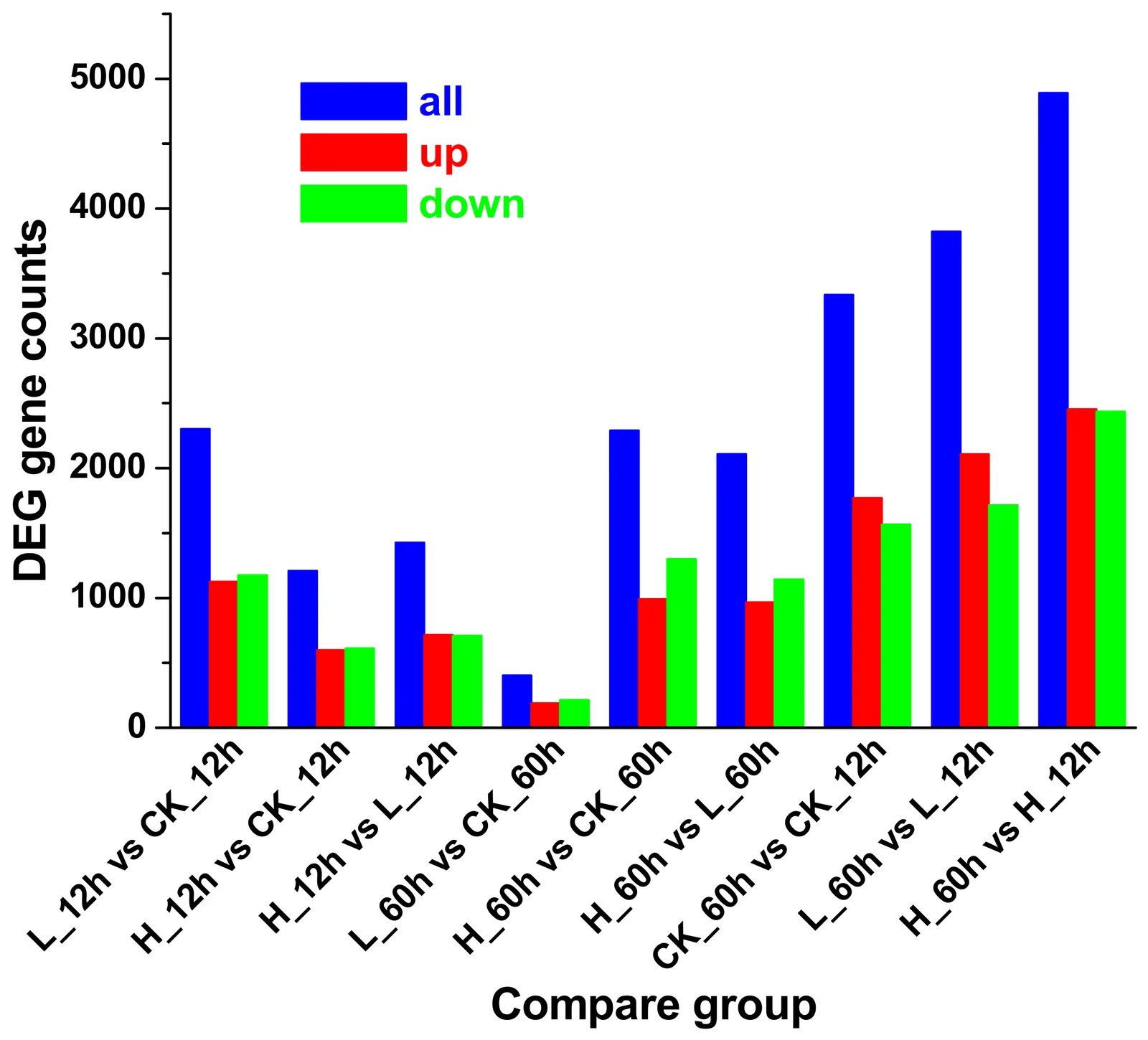
Summary of DEGs of 9 groups. Note: L: xylose ethanol low-yield strain 12.1; H: xylose ethanol high-yield strain 31.1; CK: control strain 1960; all: the total number of DEGs; up: the number of up-regulated genes; down: the number of down-regulated genes.

For the differentially expressed genes (DEGs) in the nine distinct groups, the corresponding volcano plots are shown in Figures S39–S43. The results of gene expression differences between the nine groups are presented in Figure 3. Detailed results are shown in Table S17 (comparison of Groups 1–9). The analysis results are as follows.

(1) At the early growth stage (12 h), among Groups 1–3, the number of DEGs was the highest in the comparison of low-yield strain (L) vs. wild-type strain (CK) (Group 1, 2303 DEGs), followed by high-yield strain (H) vs. low-yield strain (L) (Group 3, 1425 DEGs), and the lowest in high-yield strain (H) vs. wild-type strain (CK) (Group 2, 1209 DEGs). In both Group 1 and Group 2, the number of up-regulated genes was less than that of down-regulated genes.

(2) At the stage of maximum ethanol content (60 h), there were DEGs in Groups 4–6. In contrast to the 12 h stage, the number of DEGs was the lowest in the comparison of low-yield strain (L) vs. wild-type strain (CK) (Group 4), and the highest in high-yield strain (H) vs. wild-type strain (CK) (Group 5). The number of DEGs in the comparison of high-yield strain (H) vs. low-yield strain (L) (Group 6) ranked in the middle, consistent with the result at 12 h. For the above three comparison groups, the number of up-regulated genes was less than that of down-regulated genes.

(3) Comparison of the same strain at different fermentation time points (Groups 7–9, i.e., 60 h vs. 12 h). The largest number of differentially expressed genes (DEGs) was observed in the high-yield strain 31.1 (Group 9), followed by that in the low-yield strain (Group 8), and the smallest number of DEGs was in the wild-type strain (Group 7). For the above three groups (Groups 7–9), the numbers of DEGs in all three groups were far greater than those in Groups 1–6, and the smallest number of DEGs among all nine groups was in Group 4. For the three strains, the number of up-regulated genes was greater than that of down-regulated genes.

Corresponding Venn diagrams were plotted based on the number of differentially expressed genes (DEGs) in each comparison combination (see Figures S44 and S45). The details are as follows: At 12 h and 60 h of fermentation, we have the following: The number of common DEGs between the low-yield strain and the wild-type strain was 198 (Figure S44a); the number of common DEGs between the high-yield strain and the wild-type strain was 507 (Figure S44b); and the number of common DEGs between the high-yield strain and the low-yield strain was 551 (Figure S44c). At 12 h of fermentation, we have the following: The number of common DEGs between the wild-type strain and the low-yield strain, and between the wild-type strain and the high-yield strain, was 897 (Figure S45a). At 60 h of fermentation, we have the following: The number of common DEGs between the wild-type strain and the low-yield strain, and between the wild-type strain and the high-yield strain, was 243 (Figure S45b). At 12 h of fermentation, we have the following: The number of common DEGs among the wild-type strain, low-yield strain, and high-yield strain (between each pair) was 252 (Figure S45a). At 60 h of fermentation, we have the following: The number of common DEGs among the wild-type strain, low-yield strain, and high-yield strain (between each pair) was 76 (Figure S46b). When comparing each of the three strains between 60 h and 12 h of fermentation, the number of common DEGs was 2459 (Figure S46c). Across the two time points (12 h and 60 h), the number of common DEGs in the comparisons of the high-yield strain vs. wild-type strain and low-yield strain vs. wild-type strain was 72 (Figure S47). It can be seen that the number of common DEGs between the high-yield strain and the low-yield strain, and between the high-yield strain and the wild-type strain, was always greater than that between the low-yield strain and the wild-type strain.

In this study, the differentially expressed genes (DEGs) were clustered, with the clustering between groups shown in Figure S48 and the clustering between samples shown in Figure S49. These two Figures allow for an intuitive observation of the differences in gene expression levels among different samples.

The results of GO enrichment analysis are shown in Table 4. It can be seen that there were certain differences among different comparison combinations. The key observations are as follows: For comparisons within the same category group and the same GO category, the number of differentially expressed genes (DEGs) at the 60 h fermentation stage was generally greater than that at the 12 h fermentation stage. At 12 h of fermentation, the number of DEGs was the highest in the comparison between the wild-type strain (CK) and the low-yield strain (L), followed by that between the high-yield strain (H) and the low-yield strain (L), and the lowest in the comparison between the high-yield strain (H) and the wild-type strain (CK). At 60 h of fermentation, in contrast to the 12 h stage, the number of DEGs was the highest in the comparison between the high-yield strain (H) and the wild-type strain (CK), followed by that between the high-yield strain (H) and the low-yield strain (L), and the lowest in the comparison between the wild-type strain (CK) and the low-yield strain (L).

The results of KEGG enrichment analysis for the nine group comparisons are shown in Table 5. At the early fermentation stage (12 h), *S. stipitis* cells were mainly in the growth and reproduction phase. The total number of differentially expressed genes (DEGs) among the three strains was generally less than that at the vigorous alcoholic fermentation stage (60 h), with the exception of the comparison between the low-yield strain and the wild-type strain: the number of DEGs at 12 h (725) was significantly more than that at 60 h (104). Among the nine group comparisons, the smallest total number of DEGs was observed in the comparison between the low-yield strain and the wild-type strain at 60 h (104), while the largest was in the comparison of the high-yield strain between 60 h and 12 h (1573). This may be one of the reasons for the high xylose ethanol production of the high-yield strain 31.1. In addition, six typical combinations were selected for further analysis, and the KEGG pathways corresponding to the differentially expressed genes were listed respectively. The specific analysis results are as follows.

(1) At 12 h of fermentation, in the comparison between the high-yield strain and the wild-type strain, a total of 87 such pathways were identified, but none of them were significant. In the comparison between the high-yield strain and the low-yield strain, there were 89 such pathways in total, slightly more than the 87 pathways in the high-yield vs. wild-type comparison, among which five were significant; in the comparison between the wild-type strain and the low-yield strain, there were 89 such pathways in total, with only 1 significant one: Ribosome.

(2) At 60 h of fermentation, in the comparison between the high-yield strain and the wild-type strain, there were 90 such pathways in total, slightly more than that at 12 h (87), with only 1 significant one: Oxidative phosphorylation; in the comparison between the high-yield strain and the low-yield strain, there were 90 such pathways in total, with only 1 significant one; in the comparison between the wild-type strain and the low-yield strain, there were 78 such pathways in total, with 10 significant ones.

Based on the comparative analysis results of transcriptome sequencing, approximately 10 up-regulated genes and 10 down-regulated genes were randomly selected for qPCR validation, including key genes in key metabolic pathways (e.g., key genes in the xylose ethanol metabolism pathway: xylose reductase gene *XYL1*, xylitol dehydrogenase gene *XYL2*, xylulokinase gene *XKS1*, alcohol dehydrogenase gene *ADH4*, pyruvate decarboxylase gene, etc.).

**Figure 4:**
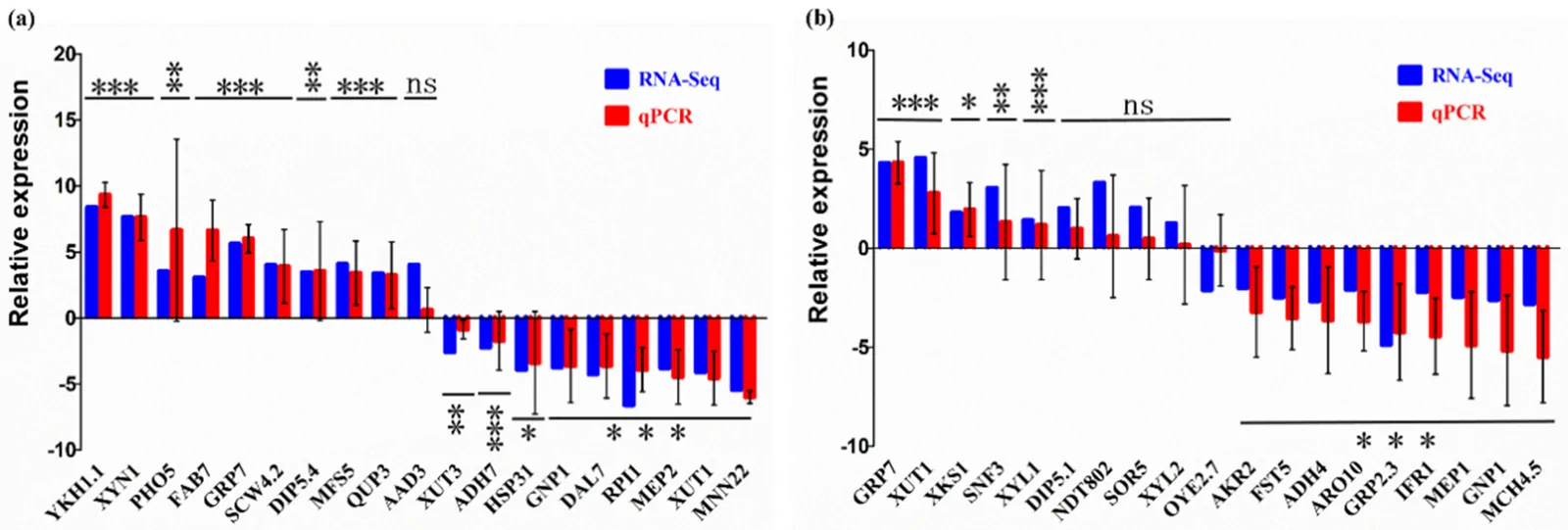
Comparison of the results of qPCR and RNA-Seq. Note: (**a**): H_12 h vs. CK_12 h; (**b**): H_12 h vs. L_12 h (H: high-yield strain 31.1; CK: the control strain 1960; L: low-yield strain 12.1; 12 h: cultivated in 15%FM at 30 °C, 130 rpm for fermentation for 12 h); the results were average and standard deviation of triplicate determination; asterisks represent significant difference among samples at *p* < 0.001 (three asterisks, highly significant) or *p* < 0.01 (two asterisks, very significant) or *p* < 0.05 (one asterisk, significant), respectively, by Student’s two-tailed *t*-test; ns: not significant, *p* ≥ 0.05.

**Figure 5:**
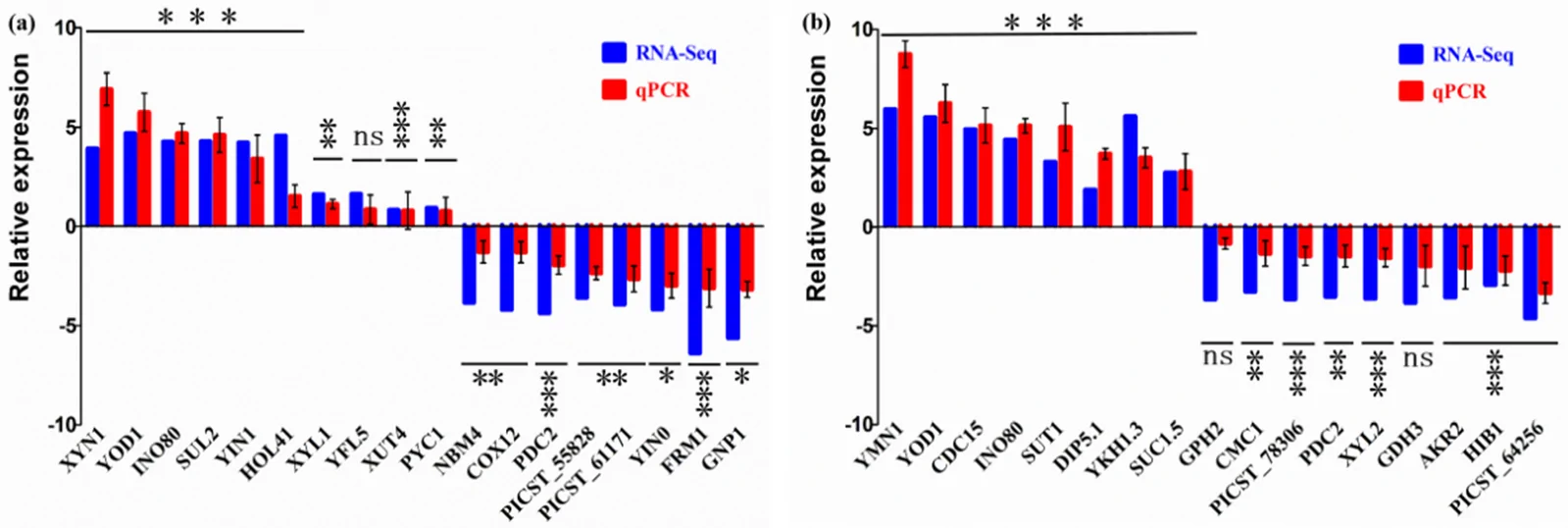
Comparison of the results of qPCR and RNA-Seq (H_60 h vs. CK_60 h). Note: (**a**): H_60 h vs. CK_60 h; (**b**): H_60 h vs. L_60 h (H: high-yield strain 31.1; CK: the control strain 1960; L: low-yield strain 12.1; 60 h: cultivated in 15%FM at 30 °C, 130 rpm for ethanol fermentation for 60 h); the results were average and standard deviation of triplicate determination; asterisks represent significant difference among samples at *p* < 0.001 (three asterisks, highly significant) or *p* < 0.01 (two asterisks, very significant) or *p* < 0.05 (one asterisk, significant), respectively, by Student’s two-tailed *t*-test; ns: not significant, *p* ≥ 0.05.

The results shown in Figure 4 and Figure 5 are the mean values and standard deviations of three parallel experiments conducted on the basis of three biological replicates for each sample (for which the corresponding total RNA was used as the qPCR template, consistent with the total RNA used for library construction). The reference gene used in all four validation groups was TAL1. The results are as follows.

(1) H_12 h vs. CK_12 h (Figure 4a): The qPCR results of the 19 verified genes were all consistent with the transcriptome sequencing analysis results. Except for the AAD3 gene (which was not significant), the other 18 genes were significant. Among them, the genes with highly significant differential expression included the sugar transport gene *XUT1*, the hexose transporter gene MFS5, and the alcohol dehydrogenase gene ADH7; the sugar transport gene XUT3 showed extremely significant differential expression. The results indicated that the accuracy of the transcriptome sequencing data reached 100%, and the data were reliable.

(2) H_12 h vs. L_12 h (Figure 4b): The qPCR results of the 19 verified genes were all consistent with the transcriptome sequencing analysis results. Among them, five genes showed no significant differential expression (including the xylitol dehydrogenase gene *XYL2* and the NADPH dehydrogenase gene OYE2.7), and 14 genes were significant. These significant genes included the xylose reductase gene *XYL1*, the xylulokinase gene *XKS1*, the high-affinity sugar transport gene *XUT1* (highly significant), the high-affinity glucose transporter gene SNF3 (extremely significant), the alcohol dehydrogenase gene *ADH4* (highly significant), and the pyruvate decarboxylase gene *ARO10* (highly significant). The results indicated that the accuracy of the transcriptome sequencing data reached 100%, and the data were reliable.

(3) H_60 h vs. CK_60 h (Figure 5a): For the qPCR results of the 18 verified genes, the up-regulated or down-regulated expression of all genes was consistent with the transcriptome sequencing analysis results. Except for the YFL5 gene (which was not significant), the other 17 genes were significant. Among them, the genes with highly significant differential expression included the high-affinity xylose transporter gene *XUT4* and the pyruvate decarboxylase gene *PDC2*; the xylose reductase gene *XYL1* showed extremely significant differential expression. The results indicated that the accuracy of the transcriptome sequencing data reached 100%, and the data were reliable.

(4) H_60 h vs. L_60 h (Figure 5b): The qPCR results of the 17 verified genes were all consistent with the transcriptome sequencing analysis results. Except for genes GPH2 and GDH3 (which were not significant), the other 15 genes were significant. Among them, the genes with highly significant differential expression included the xylitol dehydrogenase gene *XYL2* and the hexose transporter gene SUT1; the pyruvate decarboxylase gene *PDC2* showed extremely significant differential expression. The results indicated that the accuracy of the transcriptome sequencing data reached 100%, and the data were reliable.

These results indicated that the accuracy of the transcriptome sequencing results for all four groups reached 100%, and the data were reliable.

The KEGG significantly enriched metabolic pathway among the differentially expressed genes (DEGs) between the high-yield strain 31.1 and the wild-type strain 1960 was mainly oxidative phosphorylation. There were 46 DEGs with significant differential expression in total, among which only one gene was up-regulated: the NBM8 gene. Its expression product is the B18 subunit of NADH-ubiquinone oxidoreductase, which is involved in catalyzing the transfer of electrons from NADH to ubiquinone, accompanied by energy generation. In contrast, 45 genes were down-regulated, as shown in Table 6. In *S. stipitis*, a Crabtree-negative yeast, fermentation is not triggered by high sugar concentrations but rather by a decrease in oxygen availability [54]. Under microaerobic conditions, reduced respiratory activity shifts the metabolic balance toward fermentation, which favors ethanol production [55]. Therefore, the widespread down-regulation of oxidative phosphorylation genes in strain 31.1 likely reflects a metabolic shift from respiration-dependent energy generation toward a more fermentative mode, redirecting carbon flux from xylose to ethanol and contributing to the higher ethanol productivity observed in this strain.

The high-yield strain has advantages such as high ethanol production. Its advantages at the transcriptome level are as follows.

(1) At 12 h of xylose ethanol fermentation, in the comparison between the high-yield strain and the low-yield strain, the three key genes in the xylose ethanol metabolic pathway, namely xylose reductase gene *XYL1* (+1.95), xylitol dehydrogenase gene *XYL2* (+0.48), and xylulokinase gene *XKS1* (+1.32), were all up-regulated.

(2) At 60 h of fermentation, when ethanol content was the highest, regarding differentially expressed genes (DEGs), the number of DEGs was the highest in the comparison of high-yield strain vs. wild-type strain, followed by that in high-yield strain vs. low-yield strain, while the number was the lowest in low-yield strain vs. wild-type strain. In addition, in the comparison between the high-yield strain and the wild-type strain, two key genes—xylose reductase gene *XYL1* (+1.13) and high-affinity xylose transporter gene *XUT4* (+0.85)—were both up-regulated.

(3) In the comparison of the same strain at different fermentation time points (i.e., 60 h vs. 12 h), the high-yield strain 31.1 had the largest number of differentially expressed genes. Moreover, several key genes in the xylose ethanol metabolic pathway were all up-regulated, including *XYL1* (+0.05), *XKS1* (+0.86), high-affinity xylose transporter gene *XUT4* (+4.57), and pyruvate decarboxylase genes *PDC1* (+5.56), *PDC2* (+0.84), *ARO10* (*PDC6*, *PDC3*) (+9.81), and OYE2.7 (+11.41).

Preliminary revelation of the reasons for high xylose ethanol production in the high-yield strain at genomic and transcriptomic levels.

At the genomic level, there were 271 genomic structural variations. At the transcriptomic level, most genes involved in secondary metabolite synthesis, antibiotic synthesis, the ribosome pathway, amino acid biosynthesis, and the oxidative phosphorylation pathway were down-regulated; in addition, two key genes—namely the xylose reductase gene *XYL1* and the high-affinity xylose transporter gene *XUT4*—were both up-regulated.

In this study, a total of 60 candidate key genes were identified in *S. stipitis* through transcriptomic analysis. Based on their functional characteristics, these genes are presented in two separate Tables: Table 7A lists 18 candidates mainly involved in the cell cycle pathway, and Table 7B lists 42 candidates related to oxidative phosphorylation. All these candidate genes were directly identified from the transcriptomic data of the present study, providing targets for future functional validation in xylose-to-ethanol fermentation in *S. stipitis*.

## 4. Discussion

### 4.1. Analysis of Related Studies on Mutagenesis Breeding of S. stipitis: Mutagenic Improvement of S. stipitis Strains

Since *S. stipitis* strain CICC1960 is haploid, only three strains of *S. stipitis* with published genomic information are available. To the best of our knowledge, no comparative genomics study has been reported that directly compares mutagenized industrial strains of *S. stipitis* (e.g., high- and low-yielding variants derived from ^60^Co mutagenesis and long-term domestication) to elucidate the genomic basis of enhanced xylose-to-ethanol fermentation. Therefore, this study selected ^60^Co mutagenesis to treat *S. stipitis* strain CICC1960.

Mutagenesis breeding remains a feasible approach to enhance the xylose-ethanol fermentation performance of *S. stipitis* strains, and there have been several relevant reports. For instance, we have the following: Grabek et al. [53] used ultraviolet (UV) mutagenesis to screen Hansenula polymorpha strains and *S. stipitis*; Watanabe et al. [31] employed UV mutagenesis to screen *S. stipitis* strain NBRC1687; and Ren Jia et al. [27] used ^60^Co mutagenesis to screen Pachysolen tannophilus for xylose-to-ethanol fermentation. In this study, the original *S. stipitis* strain 1960 was mutagenized with nine doses of ^60^Co. A total of 100 colonies mutagenized with different doses were selected for xylose-ethanol fermentation, and a series of high-yield yeast strains as well as some low-yield strains were obtained through primary screening and secondary screening. Furthermore, a superior high-yield strain 31.1 was developed by subjecting the high-yield strains to long-term acclimation with sugarcane molasses. The ethanol production of strain 31.1 was 14.13% higher than that of the original strain. In contrast, Watanabe et al. [31] obtained the mutant strain PXF58 by mutagenizing *S. stipitis* strain NBRC1687 with UV radiation, and the increase rate of ethanol content reached 38.71%. However, a direct comparison of the improvement rates between the two studies should be made with caution. The two studies used different parental strains (CICC1960 vs. NBRC1687), different xylose concentrations (150 g/L vs. 114 g/L), and different screening and domestication protocols. Therefore, the observed difference cannot be simply attributed to the mutagenic agent alone. Mechanistically, UV radiation primarily induces point mutations (e.g., pyrimidine dimers), whereas ^60^Co γ-irradiation tends to cause DNA double-strand breaks and larger structural variations. Whether these different mutational spectra lead to systematically different outcomes for xylose-fermenting phenotypes remains an open question and would require controlled comparative studies under identical conditions. Additionally, this study also reports the low-yield strain 12.1. To date, no study has reported low-yield strains derived from *S. stipitis* mutagenesis. The ethanol production of strain 12.1 was nearly one-fourth lower than that of strain 1960, while the ethanol production of the high-yield strain 31.1 was 1.53 times that of strain 12.1.

High-yield and low-yield strains of *S. stipitis* were obtained via ^60^Co mutagenesis. When cultured in YPX medium and 15% (*w*/*v*) xylose fermentation medium, the growth rates of both mutant strains were slower than that of the original strain. Notably, when the low-yield strain fermented ethanol in the aforementioned xylose fermentation medium, its growth rate was the slowest—showing a significant difference from the original strain. In addition, both its ethanol fermentation performance and xylose utilization efficiency were the lowest, with significant differences observed when compared to the high-yield strain. The three key enzyme genes in the xylose-ethanol metabolic pathway of *S. stipitis* include the xylose reductase gene (*XYL1*), xylitol dehydrogenase gene (*XYL2*), and xylulokinase gene (*XKS1*) [56]. Zhang Lingyan et al. [15,57] overexpressed the xylitol dehydrogenase gene (*XYL2*) and found that ethanol production was increased. This study used ^60^Co to mutagenize *S. stipitis*. Based on differences in xylose-ethanol fermentation performance among different mutant strains, it proposes that ^60^Co mutagenesis causes base mutations in DNA fragments on yeast cell chromosomes, resulting in three outcomes: Positive mutations (e.g., high-yield strain 31.1); negative mutations (e.g., low-yield strain 12.1); and nonsense mutations. This study further speculates the following: (1) The base changes caused by positive and negative mutations may be associated with the three aforementioned key enzyme genes (*XYL1*, *XYL2*, and *XKS1*); and (2) compared with the original strain or low-yield strains, some key genes in the xylose-ethanol metabolic pathway of the high-yield strain 31.1 may be up-regulated. For instance, the ratio of xylitol dehydrogenase (encoded by *XYL2*) to xylose reductase (encoded by *XYL1*) may be higher, which could reduce xylitol accumulation and promote greater ethanol production. However, this hypothesis has not been directly validated by xylitol measurements in the present study. Future work should combine metabolite analyses (e.g., quantification of xylitol, glycerol, and other by-products) with multi-omics approaches to test these possibilities.

### 4.2. Comparison of Xylose Ethanol Fermentation Performance Between High-Yield Strain 31.1 and the Literature Reports

Among the xylose-ethanol-producing strains (including some new species) recently screened by Morais et al., *S. stipitis* exhibits relatively excellent fermentation performance, with a fermentation efficiency exceeding 82% [40]; Candida shehatae also achieves a fermentation efficiency of 78.26%, which is slightly lower than that of *S. stipitis*; in addition, *Zymomonas mobilis* A3 reaches a xylose-ethanol fermentation efficiency of 88.20% [38]. Currently, for fermentation using xylose as the substrate, the strain with relatively high ethanol concentration is *Paecilomyces* sp. NF1 (ATCC-20766). When this strain is used to ferment 150 g/L and 200 g/L xylose, respectively, the ethanol concentrations are 7.56% and 9.31%, respectively; the sugar-to-ethanol conversion rates are 0.39 g/g and 0.38 g/g, respectively; the fermentation efficiency is 75% in both cases. However, the fermentation period is excessively long (12 days and 13 days, respectively), with corresponding ethanol productivities of 0.21 g/(L·h) and 0.24 g/(L·h) [10]. *S. passalidarum* achieved the highest ethanol yield (0.44 g/g) and ethanol productivity (1.02 g/L·h) under the conditions of 100 g/L xylose and 32 °C [58]. Two types of recombinant strains were constructed via the chromosomal insertion of genes related to xylose assimilation and transport. After the evolved strains were subcultured 10 times, their performance was evaluated: strain CAT-1-XIT (pRS42K::XI) utilized 74% of D-xylose, produced 12.6 g/L ethanol, and had an ethanol yield of 0.31 g ethanol/g D-xylose [59]. Xylose metabolism genes from *S. stipites* were introduced into Saccharomyces cerevisiae under the regulation of different promoters, and RNA-seq was used to analyze the response of S. cerevisiae metabolism to the introduction of these xylose metabolism genes. The effects of overexpressing *S. stipitis* genes were as follows: compared with the “XR-pXDH” strain, overexpressing either the xylulokinase gene from *S. stipitis* or its XR/XDH (xylose reductase/xylitol dehydrogenase) gene combination significantly reduced xylitol accumulation (to 1.13 ± 0.06 g/L and 0.89 ± 0.04 g/L, respectively). Meanwhile, during the xylose utilization phase, ethanol production was significantly increased (by 196.14% and 148.50%, respectively) [60]. The industrial Saccharomyces cerevisiae strain SA-1 was engineered using CRISPR-Cas9 technology, with the redox xylose pathway from *S. stipitis* (synonym: *S. stipitis*)—encoded by the *XYL1*, *XYL2*, and *XYL3* genes—introduced into it. Under microaerobic conditions in a hemicellulosic hydrolysate medium, the evolved strain DPY06 and its parental strain SA-1 XR/XDH were evaluated. The results showed that the volumetric ethanol yield of DPY06 was significantly 35% higher than that of the parental strain [61]. Nine non-conventional yeast strains were screened for their performance in diluted hemicellulosic hydrolysate derived from birch. *S. stipitis* CBS 5773 exhibited weak growth and biomass production when consuming glucose; however, during the xylose utilization phase, it had a specific growth rate of 0.17 h^−1^ and a sugar co-consumption rate of 0.37 g/gdw·h [62].

The high-yield strain 31.1 screened in this study has a xylose-ethanol fermentation efficiency of 82.61% and an ethanol productivity of 0.78 g/(L·h), both of which are higher than those of *Paecilomyces* sp. NF1. Although the fermentation efficiency of the high-yield strain 31.1 is lower than that of the *S. stipitis* strain CBS 5576 (97.83%), its ethanol productivity is higher than that of CBS 5576—the latter has an ethanol productivity of 0.34 g/(L·h). In addition, the fermentation efficiency of strain 31.1 shows no significant difference from that of two recently reported *S. stipitis* strains, UFMG-CM-Y2303 and UFMG-CM-Y2108. However, the xylose-ethanol productivities of these two strains are 0.39 g/(L·h) and 0.40 g/(L·h), respectively, which are significantly lower than that of strain 31.1. Therefore, the xylose-ethanol fermentation performance of the high-yield strain 31.1 ranks among the advanced levels in domestic studies of the same type.

### 4.3. Analysis of Advantages of High-Yield Strain 31.1 in Genomics and Transcriptomics

The above comparative genomics analyses revealed several genomic features of the high-yield strain 31.1. The number of predicted genes in strain 31.1 (705) was slightly higher than that in the wild-type strain 1960 (676). Across 12 different databases, strain 31.1 consistently showed more annotated entries than the wild-type strain. Notably, in the CAZy database, strain 31.1 carried the largest number of glycosyltransferase genes (81), whereas both the wild-type and low-yield strains had 78 each. KEGG analysis indicated that strain 31.1 had more annotated genes in categories such as environmental information processing (signal transduction), lipid metabolism, polysaccharide biosynthesis, and carbohydrate metabolism. At the structural level, 271 genomic structural variations were identified between strain 31.1 and the wild-type strain, and 223 between strain 31.1 and the low-yield strain. These genomic differences, together with the transcriptomic changes presented below, provide a molecular basis for the superior fermentation phenotype of strain 31.1.

From the above-mentioned comparative genomics analysis, it can be observed that compared with the original strain, the high-yield strain has a significantly higher number of annotated genes—among which the number of genes encoding glycosyltransferases is also higher. In yeast cells, glycosyltransferases catalyze the attachment of activated sugars to various acceptor molecules, such as proteins, nucleic acids, oligosaccharides, lipids, and small molecules. Glycosylated products exhibit a variety of biological functions [63]. In addition, the number of genomic structural variations between the high-yield strain and the original strain, and between the high-yield strain and the low-yield strain, is 271 and 223, respectively. This may be the underlying reason for the high-yield strain exhibiting a series of superior phenotypes.

From the aforementioned comparative transcriptomic analysis, we have the following.

At the early fermentation stage (12 h), when comparing the high-yield strain with the low-yield strain, the significantly differentially expressed genes are associated with ribosomal processes, steroid biosynthesis, and pyruvate metabolism. Notably, pyruvate metabolism is a crucial step in the xylose-ethanol metabolic process [20,21]. At the peak ethanol production stage (60 h), when compared with the original strain, the high-yield strain shows significant differential expression of genes related to oxidative phosphorylation; when compared with the low-yield strain, the high-yield strain exhibits significant differential expression of genes involved in cysteine and methionine metabolism. Oxidative phosphorylation provides energy for yeast cells [64]. Cysteine and methionine are sulfur-containing amino acids. Methionine can be converted to S-adenosylmethionine (SAM), which plays key roles in multiple cellular processes including transsulfuration, methylation reactions, and redox balance [65]. Importantly, methionine supplementation has been shown to stabilize mitochondrial activity in yeast during ethanol fermentation, and SAM biosynthesis is required for stress tolerance [66,67]. Thus, the differential expression of methionine metabolism-related genes between the high- and low-yield strains observed in this study may contribute to the superior fermentation performance of strain 31.1, although the underlying mechanisms require further investigation.

In this study, *S. stipitis* original strain 1960, high-yield strain 31.1, and low-yield strain 12.1 were analyzed using comparative genomics and transcriptomics. To date, no prior studies on this specific aspect have been reported. Through this research, certain research findings were obtained; however, there are still many issues that deserve in-depth exploration and investigation.

## 5. Conclusions

### 5.1. Summary of Findings

In this study, we successfully developed a high-ethanol-producing *S. stipitis* strain 31.1 through ^60^Co mutagenesis combined with long-term sugarcane molasses acclimation. The strain achieved an ethanol productivity of 0.78 g/(L·h), a xylose-to-ethanol conversion rate of 0.38 g/g, and a fermentation efficiency of 82.61%, representing a substantial improvement over the wild-type strain. Comparative genomics revealed 271 structural variations and an increased repertoire of glycosyltransferase genes in strain 31.1, while transcriptomics showed down-regulation of oxidative phosphorylation genes and up-regulation of key xylose metabolism genes (*XYL1*, *XUT4*). Together, these findings suggest that the high-yield phenotype arises from a metabolic shift from respiration toward fermentation, coupled with enhanced cell wall integrity and stress tolerance. Importantly, this work provides a multi-omics resource for understanding the genetic basis of xylose fermentation in *S. stipitis* and identifies 60 candidate genes (e.g., *CDC15*, *PHO81*, and oxidative phosphorylation components) that can be prioritized for future metabolic engineering. Our study demonstrates that traditional mutagenesis, when combined with comparative genomics and transcriptomics, remains a powerful approach for strain improvement and mechanistic discovery, offering a blueprint for developing industrial yeasts capable of converting lignocellulosic sugars into biofuels.

### 5.2. Main Innovation Points

A *S. stipitis* strain 31.1 was screened and obtained via ^60^Co mutagenesis combined with sugarcane molasses acclimation, and its xylose-ethanol productivity, sugar-to-ethanol conversion rate, and fermentation efficiency were 0.78 g/(L·h), 0.38 g/g, and 82.61%, respectively. This study lays a foundation for the yeast-based conversion of bagasse to ethanol.

### 5.3. Prospects: Functional Verification of Key Genes for High Xylose-Ethanol Production and Strain Improvement in S. stipitis

Although *S. stipitis* possesses xylose fermentation capability, it faces barriers to genetic manipulation, including difficulty in genetic transformation, limited antibiotic resistance markers, and a distinct genetic code compared to most organisms. To address these issues, a series of molecular tools tailored for this yeast have been developed, including BLINCAR (a Cas9-driven bioluminescent indicator for recombination incompetence). This tool can be repeatedly used to integrate different exogenous DNA fragments into the genome of wild-type *S. stipitis* or knock out multiple endogenous genes; it also enables the addition or removal of multiple genetic elements in the wild-type *S. stipitis* genome, thereby expanding the application potential of this yeast [68]. For example, based on the candidate genes identified in this study (such as *CDC15* and *PHO81*), BLINCAR could be applied to knock out or overexpress these genes to further improve xylose fermentation efficiency or stress tolerance in non-detoxified hydrolysates. Advanced metabolic engineering research on *S. stipitis* is still limited by the lack of systems and synthetic biology approaches—such as mature in silico models, well-characterized genetic elements, and optimized genome editing tools. In the future, the integration of systems and synthetic biology strategies (e.g., combining BLINCAR with synthetic promoters or biosensors) will also help broaden the application prospects of non-conventional yeasts like *S. stipitis* in industrial biotechnology [69].

This study conducted comparative genomics and transcriptomics analyses of the high-yield strain, low-yield strain, and original strain, uncovering a wealth of important information. It preliminarily revealed the reasons for the high xylose-ethanol productivity of the high-yield strain at the genomic and transcriptomic levels. However, the research still lacks a systematic framework and depth. For this reason, future work should focus on verifying the functions of key genes involved in high xylose-ethanol production in *S. stipitis*, including techniques such as gene knockout and gene overexpression, and conduct strain improvement based on these verification results.

## Figures and Tables

**Table 2:** Statistical results of data filtering.

Sample	Insert Size (bp)	Clean Reads Length (bp)	Raw Data (Mb)	Filtered Reads (%)	Clean Data (Mb)	Clean Data GC (%)	Clean Data Q20 (%)	Clean Data Q30 (%)
1960.1	350	(150:150)	1928	13.25	1672	40.76	97.65	93.17
1960.2	350	(150:150)	2268	14.51	1939	40.75	97.60	93.07
1960.3	350	(150:150)	2260	12.64	1975	41.30	97.19	92.13
31.1.1	350	(150:150)	1981	10.91	1765	40.85	97.22	92.24
31.1.2	350	(150:150)	2466	12.74	2152	40.70	97.35	92.51
31.1.3	350	(150:150)	2083	13.50	1802	40.60	97.72	93.38
12.1.1	350	(150:150)	1700	10.86	1516	40.85	97.19	92.04
12.1.2	350	(150:150)	2420	11.36	2145	40.83	97.35	92.51
12.1.3	350	(150:150)	2716	11.96	2391	40.86	97.69	93.31

Note: The last digits 1–3 of sample names represent 3 biological replicates; insert size (bp): Length of the library insert fragment; Clean Reads Length (bp): Read lengths of Read1 and Read2; Raw Data (Mb): Volume of raw data; Clean Data (Mb): Volume of data after processing; Filtered Reads (%): Proportion of filtered reads in the total number of reads in the raw data (%); Clean Data GC (%): GC content of the clean data; Clean Data Q20 (%): Q20 quality score of the clean data; Clean Data Q30 (%): Q30 quality score of the clean data.

**Table 3:** Coverage statistics of samples compared to reference sequences.

Query Name *	avg_depth	Coverage ≥ 1×	Coverage ≥ 4×	Coverage ≥ 10×	Coverage ≥ 20×	Map Rate	Mismatch Rate	Total Base	Total Map Base	Total Mismatch
1960.1	102	100	100	99.99	99.98	94.00	0.22	1,672,367,400	1,572,099,075	3,455,175
1960.2	118	100	99.99	99.99	99.99	93.73	0.23	1,938,782,700	1,817,129,942	4,115,758
1960.3	120	100	100	99.99	99.99	93.83	0.28	1,974,672,900	1,852,785,330	5,238,120
31.1.1	111	100	100	99.99	99.99	96.72	0.25	1,764,929,400	1,707,078,004	4,255,496
31.1.2	134	100	99.99	99.99	99.99	96.23	0.25	2,152,038,300	2,070,981,876	5,214,774
31.1.3	112	100	99.99	99.99	99.98	95.63	0.21	1,802,120,100	1,723,444,480	3,596,570
12.1.1	95	100	99.99	99.99	99.98	96.53	0.29	1,515,646,200	1,463,047,565	4,272,235
12.1.2	134	100	100	99.99	99.99	96.46	0.23	2,144,779,500	2,068,865,543	4,734,457
12.1.3	150	100	100	99.99	99.99	96.83	0.21	2,391,256,800	2,315,438,823	4,920,177

Note: *: Reference genome ID; database ID of *S. stipitis* CBS 6054 is GCA_000209165.1; Query name: Target sample name; avg depth Reads: Average sequencing depth (Reads); Coverage ≥ 1×, Coverage ≥ 4×, Coverage ≥ 10×, Coverage ≥ 20×: Percentage of the reference genome region with coverage ≥ 1×, 4×, 10×, and 20×, respectively (%); Map rate: Mapping rate (%); Mismatch rate: Percentage of sites with mismatches in the aligned region (%); Total base: Total number of bases in Reads; Total map base: Total number of mapped bases in Reads; Total mismatch: Total number of mismatch sites in alignment.

**Table 4:** GO analysis of DEGs of 9 groups.

Serial No.	Combination	Biological Process (BP)			Cellular Component (CC)			Molecular Function (MF)		
		Number of Differentially Expressed Genes (DEGs)	Number of Up-Regulated Genes	Number of Down-Regulated Genes	Number of Differentially Expressed Genes (DEGs)	Number of Up-Regulated Genes	Number of Down-Regulated Genes	Number of Differentially Expressed Genes (DEGs)	Number of Up-Regulated Genes	Number of Down-Regulated Genes
1	H_12 h vs. CK_12 h	459	224	235	215	111	104	516	244	272
2	H_12 h vs. L_12 h	564	276	288	271	146	125	695	344	351
3	L_12 h vs. CK_12 h	908	452	456	450	218	232	1067	504	563
4	H_60 h vs. CK_60 h	805	340	465	397	172	225	1026	421	605
5	H_60 h vs. L_60 h	765	352	413	363	162	201	973	429	544
6	L_60 h vs. CK_60 h	169	84	85	77	32	45	178	87	91
7	H_60 h vs. H_12 h	1791	916	875	861	433	428	2257	1070	1187
8	CK_60 h vs. CK_12 h	1257	664	593	609	324	285	1556	761	795
9	L_60 h vs. L_12 h	1425	781	644	690	377	313	1755	911	844

**Table 5:** KEGG analysis of DEGs of 9 groups.

Serial No.	Comparison Combination	Total Number of Differentially Expressed Genes	Number of Up-Regulated Genes	Number of Down-Regulated Genes
1	H_12 h vs. CK_12 h	325	150	175
2	H_12 h vs. L_12 h	471	259	212
3	L_12 h vs. CK_12 h	725	306	419
4	H_60 h vs. CK_60 h	736	299	437
5	H_60 h vs. L_60 h	629	273	356
6	L_60 h vs. CK_60 h	104	54	50
7	H_60 h vs. H_12 h	1573	667	906
8	CK_60 h vs. CK_12 h	1068	437	631
9	L_60 h vs. L_12 h	1232	554	678

Note: H: High-yield strain 31.1; CK: Wild-type strain 1960; L: Low-yield strain 12.1.

**Table 6:** The significantly enriched KEGG pathways of DEGs between 31.1 and 1960.

Gene Name	Description	*p*adj	Log_2_ (Fold Change)
*COR1*	Ubiquinol−cytochrome c reductase core subunit 1	4.33 × 10^−34^	−3.52
*SDH1*	Succinate dehydrogenase	2.68 × 10^−24^	−2.00
*VMA5*	Vacuolar ATP synthase subunit C	2.38 × 10^−16^	−1.68
*PICST_79236*	Predicted protein	3.99 × 10^−13^	−1.37
*NUO20*	NADH-ubiquinone oxidoreductase	4.58 × 10^−7^	−2.14
*COX4*	Cytochrome c oxidase polypeptide mitochondrial precursor	1.31 × 10^−6^	−3.28
*NBM8*	NADH-ubiquinone oxidoreductase B18 subunit	4.21 × 10^−6^	+1.94
*UQC2*	Ubiquinol-cytochrome-c reductase complex core protein mitochondrial precursor	4.87 × 10^−6^	−1.92
*COX10*	Heme A farnesyltransferase	8.71 × 10^−6^	−1.38
*ATP1*	F1F0-ATPase complex alpha subunit	8.91 × 10^−6^	−2.16
*PICST_90638*	predicted protein	9.25 × 10^−6^	−3.01
*ATP16*	ATP synthase delta subunit	1.07 × 10^−5^	−2.88
*COX11*	Cytochrome oxidase assembly factor COX11	1.60 × 10^−5^	−2.33
*COX12*	Cytochrome c oxidase polypeptide vib	4.29 × 10^−5^	−4.19
*SDH4*	Succinate dehydrogenase membrane anchor subunit	6.16 × 10^−5^	−2.64
*NBM4*	NADH-ubiquinone oxidoreductase B14 subunit	1.75 × 10^−5^	−3.84
*QCR6*	Ubiquinol-cytochrome c oxidoreductase subunit 6	1.76 × 10^−5^	−2.86
*NUO30*	NADH-ubiquinone oxidoreductase	1.68 × 10^−4^	−2.58
*ATP17*	ATP synthase f chain mitochondrial precursor	2.01 × 10^−4^	−2.82
*QCR10*	Subunit of the ubiqunol-cytochrome c oxidoreductase complex	2.28 × 10^−4^	−2.44
*ATP5*	F1F0-ATPase subunit	4.20 × 10^−5^	−3.60
*COX15*	Cytochrome c oxidase assembly protein COX15	2.63 × 10^−4^	−1.68
*COX6*	Subunit VI of cytochrome c oxidase	4.55 × 10^−4^	−3.58
*CYT1*	Cytochrome c1	4.68 × 10^−4^	−2.56
*COX13*	Cytochrome c oxidase subunit VIa	4.96 × 10^−4^	−2.75
*PICST_82538*	Predicted protein	6.59 × 10^−4^	−3.44
*ATP3*	ATP synthase gamma subunit	9.76 × 10^−4^	−2.29
*UCRI*	Ubiquinol-cytochrome c reductase iron–sulfur subunit mitochondrial precursor	1.10 × 10^−3^	−1.68
*QCR8*	Ubiquinol-cytochrome-c reductase chain VIII	1.27 × 10^−3^	−2.08
*ATP14*	F1F0-ATPase complex subunit h	1.63 × 10^−3^	−2.07
*NUO10*	NADH dehydrogenase (ubiquinone) 1 alpha	1.81 × 10^−3^	−2.84
*NUC1*	NADH-ubiquinone oxidoreductase 49 kDa subunit mitochondrial precursor	2.19 × 10^−3^	−1.82
*NUO21.2*	NADH-ubiquinone oxidoreductase 21 kDa subunit mitochondrial precursor	2.25 × 10^−3^	−3.00
*COX7*	Cytochrome c oxidase polypeptide VII	2.57 × 10^−3^	−2.61
*VMA4*	V-ATPase E subunit	2.82 × 10^−3^	−1.81
*NUO24*	Subunit NUHM of NADH:Ubiquinone Oxidoreductase	5.99 × 10^−3^	−2.11
*ATP4*	ATP synthase F0 sector subunit 4	1.21 × 10^−2^	−2.08
*SDH6*	Succinate dehydrogenase	1.44 × 10^−2^	−1.04
*NUO13*	NADH-ubiquinone oxidoreductase	1.69 × 10^−2^	−2.13
*COX5a*	Cytochrome-c oxidase subunit Va	1.73 × 10^−2^	−2.51
*UCR7*	Ubiquinol-cytochrome c reductase complex 14 kDa protein	1.73 × 10^−2^	−1.49
*PICST_81272*	Predicted protein	2.95 × 10^−2^	−1.51
*NUO1*	Mitochondrial complex I NUIM TYKY subunit	4.42 × 10^−2^	−1.43
*VMA2*	Vacuolar ATP synthase subunit B	4.47 × 10^−2^	−1.06
*SDH5*	Membrane anchor in succinate dehydrogenase complex	4.48 × 10^−2^	−1.96
*NUO51*	NADH-ubiquinone oxidoreductase 51 kDa subunit mitochondrial precursor	4.58 × 10^−2^	−0.91

Note: *p*adj: Corrected *p*-value, when the ethanol yield reaches its maximum after 60 h of fermentation with 15% (*w*/*v*) xylose.

**Table 7:** (**A**). Cell cycle candidate key genes identified from prior comparative genomics work in *S. cerevisiae*. (**B**) Oxidative phosphorylation candidate key genes directly identified from the current transcriptomic analysis in *S. stipites*.

No.	Gene	Gene Annotation	KEGG (Pathway)
(**A**)			
1	*CDC4*	F-box and WD-40 domain protein CDC4	Cell cycle—yeast
2	*DBF2*	Cell cycle protein kinase DBF2 [EC:2.7.11.-]	Cell cycle—yeast
3	*MCM5*	DNA replication licensing factor MCM5 [EC:5.6.2.3]	Cell cycle—yeast
4	*CDC15*	Cell division control protein CDC15 [EC:2.7.11.1]	Cell cycle—yeast
5	*CDH1*	Cell division cycle 20-like protein 1, cofactor of APC complex	Cell cycle—yeast
6	*MCM7*	DNA replication licensing factor MCM7 [EC:5.6.2.3]	Cell cycle—yeast
7	*CDC6*	Cell division control protein 6	Cell cycle—yeast
8	*GRR1*	F-box and leucine-rich repeat protein GRR1	Cell cycle—yeast
9	*CLB4*	G2/mitotic-specific cyclin 3/4	Cell cycle—yeast
10	*SMC2*	Structural maintenance of chromosome 2	Cell cycle—yeast
11	*MPS1*	Serine/threonine-protein kinase TTK/MPS1 [EC:2.7.12.1]	Cell cycle—yeast
12	*CKS1*	Cyclin-dependent kinase regulatory subunit CKS1	Cell cycle—yeast
13	*PHO81*	CDK inhibitor PHO81	Cell cycle—yeast
14	*CDC27*	Anaphase-promoting complex subunit 3	Cell cycle—yeast
15	*ORC2*	Origin recognition complex subunit 2	Cell cycle—yeast
16	*CDC53*	Cullin 1	Cell cycle—yeast
17	*CDC55*	Coiled-coil domain-containing protein 55	Cell cycle—yeast
18	*BRN1*	Condensin complex subunit 2	Cell cycle—yeast
(**B**)			
1	*COR1*	Ubiquinol-cytochrome c reductase core subunit 1	Oxidative phosphorylation
2	*SDH1*	Succinate dehydrogenase	Oxidative phosphorylation
3	*VMA5*	Vacuolar ATP synthase subunit C	Oxidative phosphorylation
4	*NUO20*	NADH-ubiquinone oxidoreductase	Oxidative phosphorylation
5	*COX4*	Cytochrome c oxidase polypeptide mitochondrial precursor	Oxidative phosphorylation
6	*NBM8*	NADH-ubiquinone oxidoreductase B18 subunit	Oxidative phosphorylation
7	*UQC2*	Ubiquinol-cytochrome-c reductase complex core protein mitochondrial precursor	Oxidative phosphorylation
8	*COX10*	Heme A farnesyltransferase	Oxidative phosphorylation
9	*ATP1*	F1F0-ATPase complex alpha subunit	Oxidative phosphorylation
10	*ATP16*	ATP synthase delta subunit	Oxidative phosphorylation
11	*COX11*	Cytochrome oxidase assembly factor COX11	Oxidative phosphorylation
12	*COX12*	Cytochrome c oxidase polypeptide vib	Oxidative phosphorylation
13	*SDH4*	Succinate dehydrogenase membrane anchor subunit	Oxidative phosphorylation
14	*NBM4*	NADH-ubiquinone oxidoreductase B14 subunit	Oxidative phosphorylation
15	*QCR6*	Ubiquinol-cytochrome c oxidoreductase subunit 6	Oxidative phosphorylation
16	*NUO30*	NADH-ubiquinone oxidoreductase	Oxidative phosphorylation
17	*ATP17*	ATP synthase f chain mitochondrial precursor	Oxidative phosphorylation
18	*QCR10*	Subunit of the ubiqunol-cytochrome c oxidoreductase complex	Oxidative phosphorylation
19	*ATP5*	F1F0-ATPase subunit	Oxidative phosphorylation
20	*COX15*	Cytochrome c oxidase assembly protein COX15	Oxidative phosphorylation
21	*COX6*	Subunit VI of cytochrome c oxidase	Oxidative phosphorylation
22	*CYT1*	Cytochrome c1	Oxidative phosphorylation
23	*COX13*	Cytochrome c oxidase subunit VIa	Oxidative phosphorylation
24	*ATP3*	ATP synthase gamma subunit	Oxidative phosphorylation
25	*UCRI*	Ubiquinol-cytochrome c reductase Iron–sulfur subunit mitochondrial precursor	Oxidative phosphorylation
26	*QCR8*	Ubiquinol-cytochrome-c reductase chain VIII	Oxidative phosphorylation
27	*ATP14*	F1F0-ATPase complex subunit h	Oxidative phosphorylation
28	*NUO10*	NADH dehydrogenase (ubiquinone) 1 alpha	Oxidative phosphorylation
29	*NUC1*	NADH-ubiquinone oxidoreductase 49 kDa subunit mitochondrial precursor	Oxidative phosphorylation
30	*NUO21.2*	NADH-ubiquinone oxidoreductase 21 kDa subunit mitochondrial precursor	Oxidative phosphorylation
31	*COX7*	Cytochrome c oxidase polypeptide VII	Oxidative phosphorylation
32	*VMA4*	V-ATPase E subunit	Oxidative phosphorylation
33	*NUO24*	Subunit NUHM of NADH:Ubiquinone Oxidoreductase	Oxidative phosphorylation
34	*ATP4*	ATP synthase F0 sector subunit 4	Oxidative phosphorylation
35	*SDH6*	Succinate dehydrogenase	Oxidative phosphorylation
36	*NUO13*	NADH-ubiquinone oxidoreductase	Oxidative phosphorylation
37	*COX5a*	Cytochrome-c oxidase subunit Va	Oxidative phosphorylation
38	*UCR7*	Ubiquinol-cytochrome c reductase complex 14 kDa protein	Oxidative phosphorylation
39	*NUO1*	Mitochondrial complex I NUIM TYKY subunit	Oxidative phosphorylation
40	*VMA2*	Vacuolar ATP synthase subunit B	Oxidative phosphorylation
41	*SDH5*	Membrane anchor in succinate dehydrogenase complex	Oxidative phosphorylation
42	*NUO51*	NADH-ubiquinone oxidoreductase 51 kDa subunit mitochondrial precursor	Oxidative phosphorylation

## Data Availability

Due to intellectual property protection, all raw sequencing data have not been deposited in public databases. However, researchers may access the data upon submitting a reasonable request to the corresponding author, provided that the request is approved by the local ethics committee and fully complies with applicable data protection regulations.

## Supplementary Materials

The following supporting information can be downloaded at: https://www.mdpi.com/article/10.3390/life16081307/s1.

## Abbreviations

The following abbreviations are used in this manuscript:

YPX	Yeast extract peptone xylose broth
FPKM	Fragments per kilobase of transcript per million mapped reads
DEG(s)	Differentially expressed gene(s)
KEGG	Kyoto Encyclopedia of Genes and Genomes
PCR	Polymerase chain reaction
qPCR	Quantitative real time polymerase chain reaction
TCA	Tricarboxylic acid
