# Supplementary material for: Comparative Genomic and Transcriptomic Analyses of 60Co-Mutagenized Scheffersomyces stipitis Strains: Identification of Candidate Genes Associated with High-Ethanol-Producing Xylose-to-Ethanol Fermentation
Source: Life (Basel). 2026 Aug 10;16(8):1307. doi: 10.3390/life16081307 (PMC13514516; doi:10.3390/life16081307)
Supplement: Supplementary file 1 [file life-16-01307-s001.zip › life-4353259-supplementary.pdf]

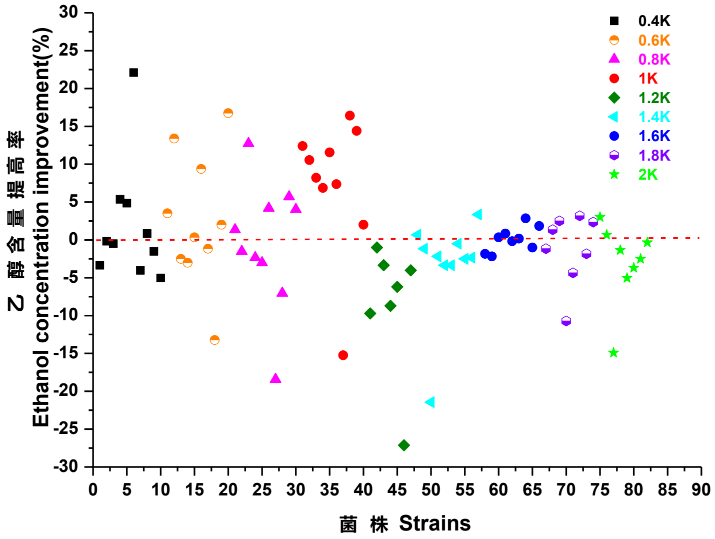

Figure S1 Comparison of screening results of ^{60}Co mutagenesis
Note: 9 dosages in total, 0.4-2.0 KGy.

Table S1 The results of repeat screening of ^{60}Co mutants

Strain	Ethanol Content (%, v/v)	Increase Rate (%)
1960（CK）	5.87 ± 0.03	-----
1K-9[a]	6.46 ± 0.02	10.05
0.4K-5	6.39 ± 0.03	8.69
1K-1	6.25 ± 0.03	6.30
1K-8	6.16 ± 0.02	4.77
1K-5	6.24 ± 0.04	6.13
0.4K-6	6.09 ± 0.07	3.58
0.4K-4	6.16 ± 0.07	4.77
12K-1[b]	4.47 ± 0.09	-23.83

Note: Fermentation conditions: 30 °C, 130 rpm, 84 h; a: Strain 1K-9 is the parental strain of the high-yield strain 31.1 (the research object of this study); b: Strain 12K-1 is the alternative name of the low-yield strain 12.1 studied in this paper; Results are presented as the mean ± standard deviation of duplicate experiments.

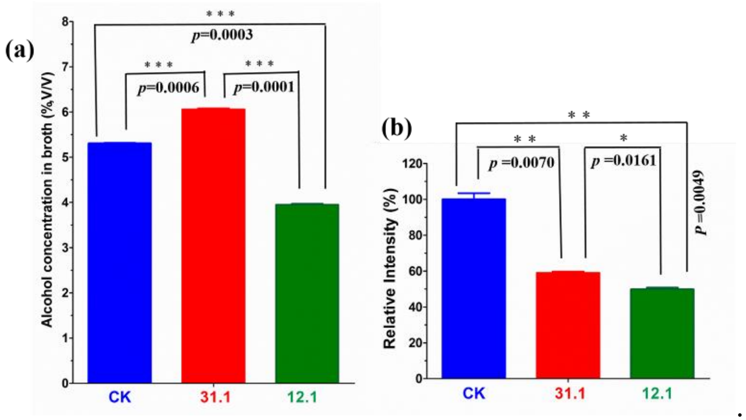

Figure S2 Comparison of ethanol production from xylose and relative respiratory intensity

Note: a: Ethanol; b: Relative respiratory intensity; The results were average and standard deviation of duplicate determination; Asterisks represent significant difference among samples at p<0.001 (three asterisks, highly significant) or p<0.01（two asterisks, very significant）or p<0.05（one asterisk, significant）respectively by Student's two-tailed t-test.

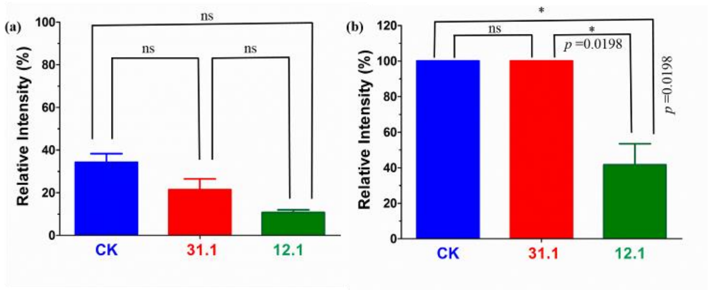

Figure S3 The difference of gas production

Note: a: Incubated at 30 ℃ for 48 h in YPX; b: Incubated at 30 ℃ for 60 h in YPX; The results were average and standard deviation of duplicate determination; One asterisk represents significant difference among samples at p<0.05; ns: not significant, p≥0.05: Differences among samples assessed by Student's t-tests was not significant.

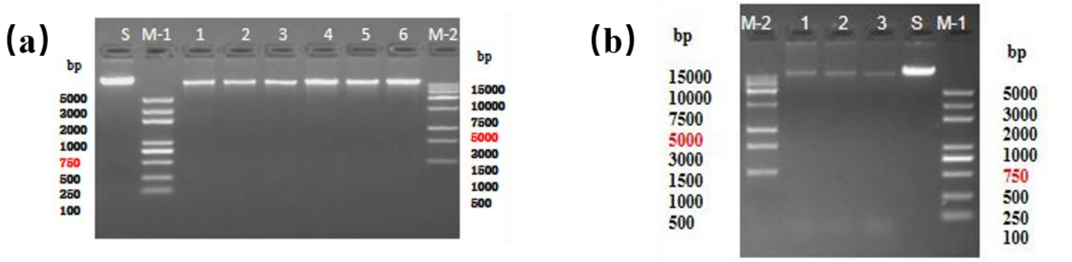

Figure S4 Genomic DNA of P. stipitis strains

Note: a: two mutant strains; Lane: 1-3: low-yield strain 12.1, sample 1 μ L in the original solution;4-6: high-yield strain 31.1, sample 1 μL in the original solution; M-1: trans 2K plus, sample 2 μL;M-2: trans 15K plus, sample 2 μL; S: standard,sample 5 μL(10 ng/μL); b: control strain 1960; Lane: 1-3: control strain 1960, sample 5 μL in the original solution; M-1: trans 2K plus, sample 2 μL; M-2:trans 15K plus, sample 2 μL; S: standard, sample 5 μL(10 ng/μL).

Table S2 Results of genomic DNA of *P. stipitis* strains

Serial No.	Sample Name*	Qubit Concentration (ng/μL)	Volume (μL)	Total Amount (μg)	Test Conclusion
1	12.1.1	21.60	38.00	0.82	A
2	12.1.2	15.80	38.00	0.60	A
3	12.1.3	22.00	38.00	0.84	A
4	31.1.1	33.20	38.00	1.26	A
5	31.1.2	33.40	38.00	1.27	A
6	31.1.3	34.80	38.00	1.32	A
7	1960.1	4.28	95.00	0.41	A
8	1960.2	3.76	95.00	0.36	A
9	1960.3	3.24	95.00	0.31	A

Note: Explanation of sample names: Low-yield strain 12.1; High-yield strain 31.1; Wild-type strain 1960; The last digits (1, 2, 3) represent 3 biological replicates; A: Indicates that the total DNA extracted from *Pichia stipitis* strain samples is of satisfactory quality, which allows proceeding to the next step of library construction.

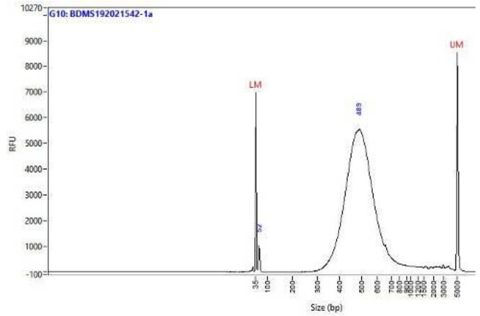

Figure S5 Detection result of genomic DNA of low-yield strain 12.1.1

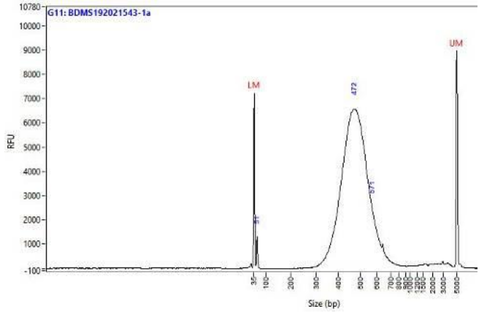

Figure S6 Detection result of genomic DNA of low-yield strain 12.1.2

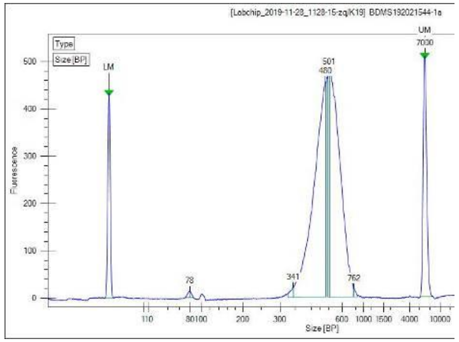

Figure S7 Detection result of genomic DNA of low-yield strain 12.1.3

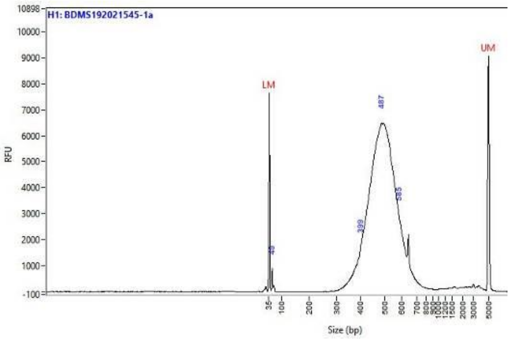

Figure S8 Detection result of genomic DNA of high-yield strain 31.1.1

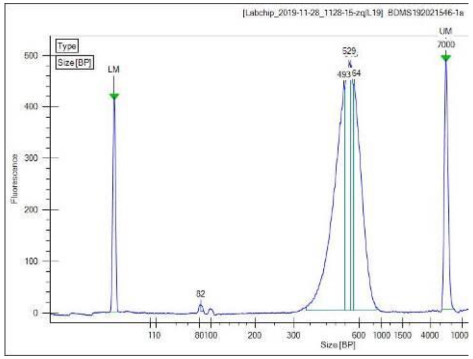

Figure S9 Detection result of genomic DNA of high-yield strain 31.1.2

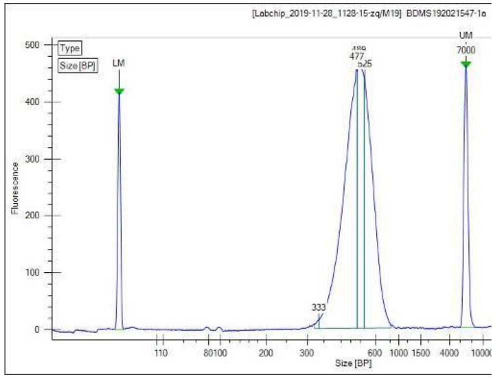

Figure S10 Detection result of genomic DNA of high-yield strain 31.1.3

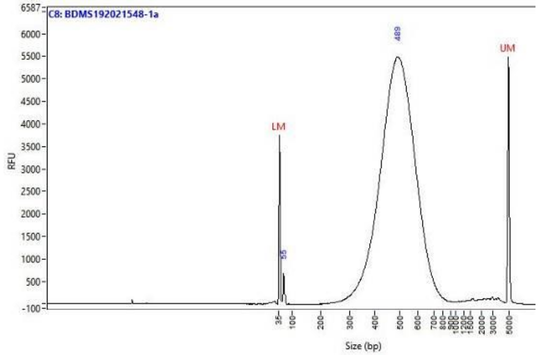

Figure S11 Detection result of genomic DNA of control strain 1960.1

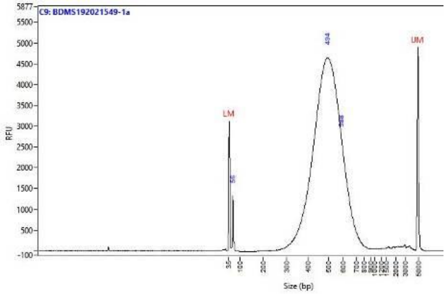

Figure S12 Detection result of genomic DNA of control strain 1960.2

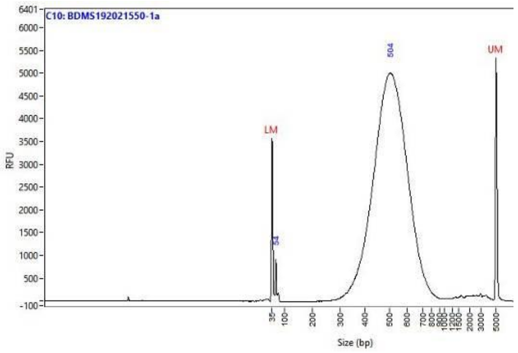

Figure S13 Detection result of genomic DNA of control strain 1960.3

Table S3 Datas of 3 strains resequencing after combined processing

Sample ID	Insert Size(bp)	Clean Reads Length(bp)	Raw Data(Mb)	Filtered Reads(%)	Clean Data(Mb)	Clean Data GC(%)	Clean Data Q20(%)	Clean Data Q30(%)
12.1	350	(150:150)	6,836	11.39	6,052	40.85	97.29	92.62
31.1	350	(150:150)	6,530	12.38	5,719	40.72	97.43	92.71
1960	350	(150:150)	6,456	13.59	5,578	40.95	97.47	92.76

Table S4 Coverage and sequencing depth statistics

Sample	Total Num(>500bp)	Total Length(bp)	N50 Length(bp)	N90 Length(bp)	Max Length(bp)	Min Length(bp)	Sequence GC%
12.1	182	14,831,711	281,459	76,420	704,212	509	41.05
31.1	194	14,835,285	257,400	73,548	705,715	509	41.05
1960	198	14,873,185	299,651	76,392	705,865	501	41.05

Note: Statistical scope: Scaffolds (scaffold sequences) with a length of over 500 bp; N50 and N90 refer to the length of the last scaffold or contig (contig

sequence) when they account for 50% and 90% of the total length respectively.

Table S5 Statistics of contigs

Sample	Total Num(>500bp)	Total Length(bp)	N50 Length(bp)	N90 Length(bp)	Max Length(bp)	Min Length(bp)	Sequence GC%
1960	249	14,872,576	299,651	70,283	705,865	207	41.05
31.1	250	14,834,702	235,421	68,926	705,715	201	41.05
12.1	241	14,831,030	254,314	74,195	673,519	215	41.05

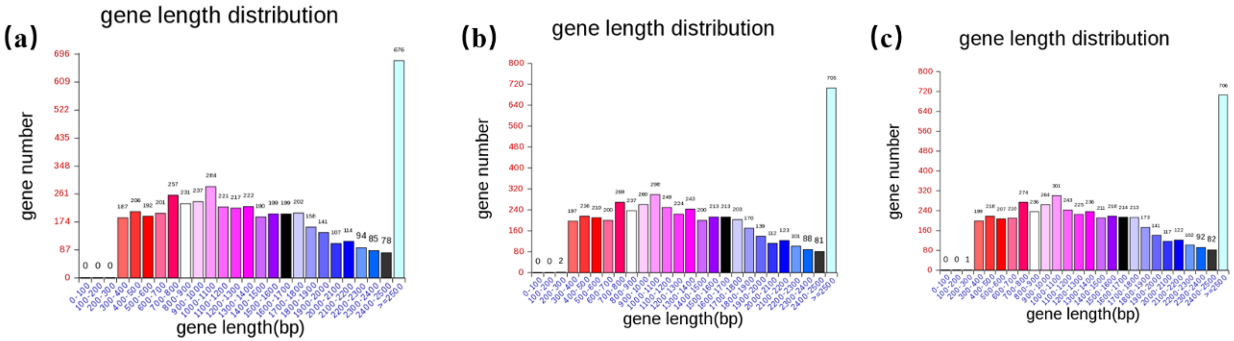

Figure S14 Gene component analysis of 3 strains

Note：a：1960；b：31.1；c：12.1.

Table S6 Comparison of gene numbers

Sample ID	Genome size(bp)	Gene number	Gene total length(bp)	Gene average length(bp)	Gene length/Genome(%)
1960	14,873,185	4,698	7,447,987	1,585	50.08
31.1	14,835,285	4,953	7,824,852	1,580	52.74
12.1	14,831,711	5,005	7,903,175	1,579	53.29

Table S7 Comparison of ncRNA

Sample ID	Type	Number	Average length(bp)	Total length(bp)
12.1	tRNA	170	78	13,418
12.1	5s(denovo)	0	0	0
12.1	5.8s(denovo)	0	0	0

12.1	18s(denovo)	0	0	0
12.1	28s(denovo)	0	0	0
12.1	sRNA	0	0	0
12.1	snRNA	36	140	5,052
12.1	miRNA	0	0	0
1960	tRNA	171	78	13,499
1960	5s(denovo)	0	0	0
1960	5.8s(denovo)	0	0	0
1960	18s(denovo)	0	0	0
1960	28s(denovo)	0	0	0
1960	sRNA	0	0	0
1960	snRNA	36	140	5,052
1960	miRNA	0	0	0
31.1	tRNA	170	78	13,418
31.1	5s(denovo)	0	0	0
31.1	5.8s(denovo)	0	0	0
31.1	18s(denovo)	0	0	0
31.1	28s(denovo)	0	0	0
31.1	sRNA	0	0	0
31.1	snRNA	36	140	5,052
31.1	miRNA	0	0	0

Table S8 Comparison of repeat sequence

Sample ID	Type	Number	Total Length(bp)	In Genome(%)	Average length(bp)
12.1	LTR	984	80,717	0.5442	85
12.1	DNA	408	28,318	0.1909	75
12.1	LINE	495	41,325	0.2786	92
12.1	SINE	44	2,237	0.0151	53
12.1	RC	26	1,999	0.0135	77
12.1	Unknown	11	751	0.0051	68
12.1	Total	1,968	147,114	0.9919	83
1960	LTR	1,005	85,473	0.5747	87
1960	DNA	411	29,269	0.1968	76
1960	LINE	504	42,566	0.2862	92
1960	SINE	44	2,237	0.0150	53

1960	RC	26	2,029	0.0136	78
1960	Unknown	13	1,021	0.0069	79
1960	Total	2,003	154,276	1.0373	85
31.1	LTR	996	82,134	0.5536	85
31.1	DNA	410	27,943	0.1884	73
31.1	LINE	500	41,723	0.2812	93
31.1	SINE	44	2,237	0.0151	53
31.1	RC	26	2,029	0.0137	78
31.1	Unknown	11	765	0.0052	70

Table S9 Summary of database annotation results

Sample	NR	SwissProt	KEGG	KOG	TCDB	GO	PHI	DFVF	P450	Secretory_Protein	CAZy	Pfam
1960	4,578	2,361	4,562	1,774	346	3,365	536	312	27	146	158	3,365
31.1	4,822	2,478	4,805	1,868	366	3,537	555	327	29	151	159	3,537
12.1	4,870	2,529	4,853	1,891	370	3,588	573	333	29	151	165	3,588

Note: NR, Non-Redundant Protein Database: a non-redundant protein database; Swiss-Prot: a gene database established by the UniProt Consortium, whose annotation results have been verified by experiments with high reliability; KEGG, Kyoto Encyclopedia of Genes and Genomes: a comprehensive database, whose core sub-database is the pathway database; KOG Database (full name: EuKaryotic Orthologous Groups): a database for eukaryotic orthologs; COG, Cluster of Orthologous Groups of proteins: a protein database; TCDB, Transporter Classification Database: a transporter classification database; GO, Gene Ontology (Gene Ontology, commonly used for gene function annotation); PHI, Pathogen-Host Interactions database: a database of genes related to pathogen-host interactions; DFVF, Database of Fungal Virulence Factors: a database of fungal virulence factors; P450: Fungal Cytochrome P450 Database; Secretory Protein: Secretory Protein Database; CAZy, Carbohydrate-Active enZYmes Database: a database related to carbohydrate-active enzymes; Pfam: Protein Family Database.

Table S10 CAZy analysis

Sample ID	CBM	CE	GH	GT	PL	AA
1960	12	4	63	78	0	6
31.1	12	3	62	81	0	6
12.1	13	4	70	78	0	6

Note: CBM: Carbohydrate-Binding Modules (CBMs), i.e., domains that bind to carbohydrates; CE: Carbohydrate Esterases (CEs), ; GH: Glycoside Hydrolases (GHs) ; GT: GlycosylTransferases (GTs) ; PL: Polysaccharide Lyases (PLs) ; AA: Auxiliary Activities (AAs), i.e., enzymes with redox functions.

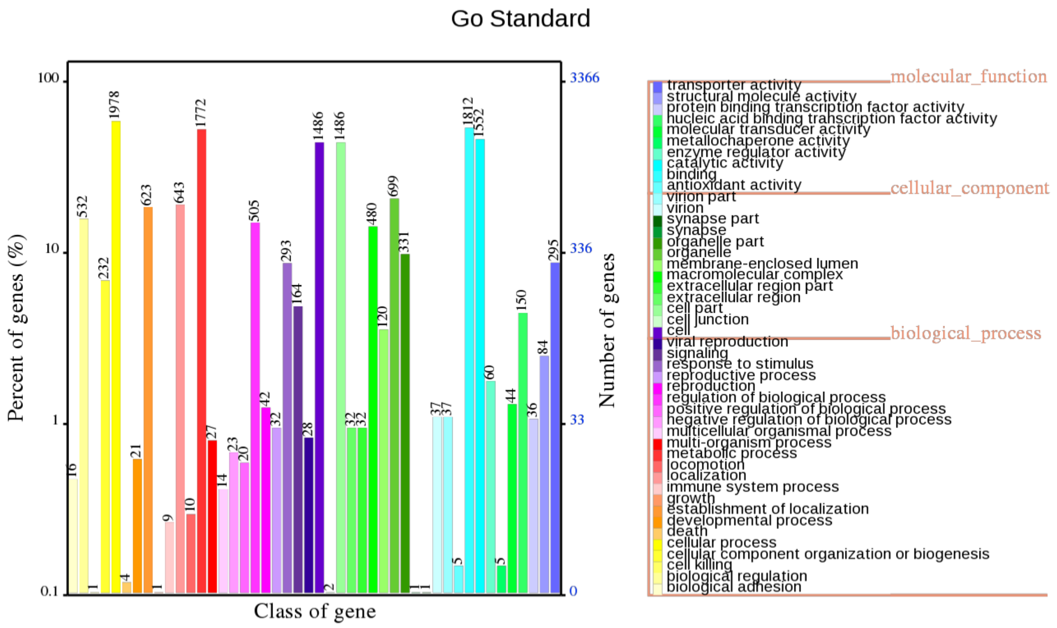

Figure S15 GO analysis of 1960

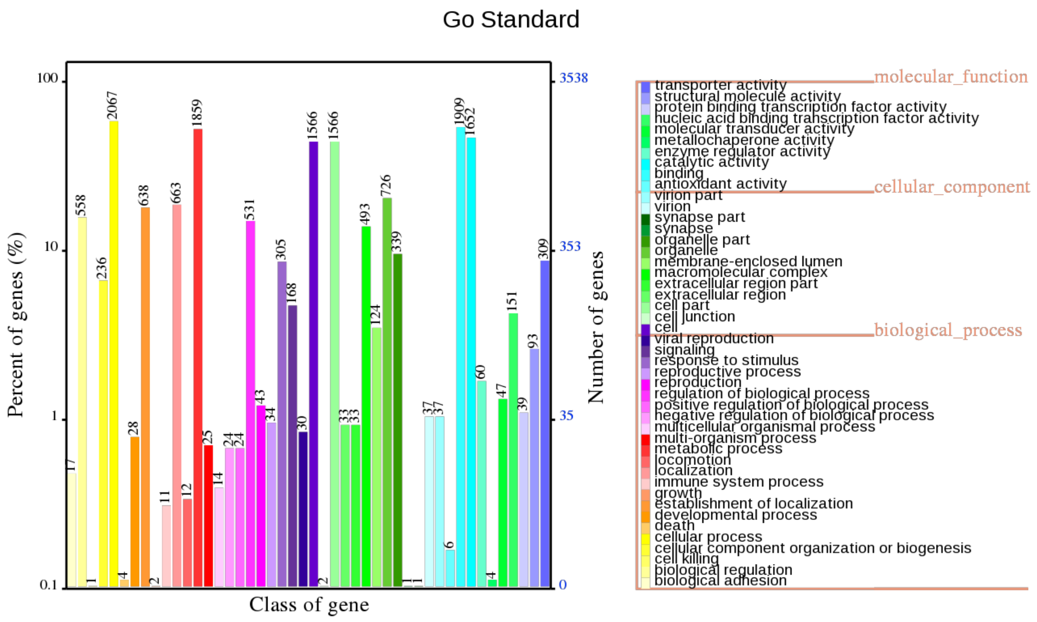

Figure S16 GO analysis of 31.1

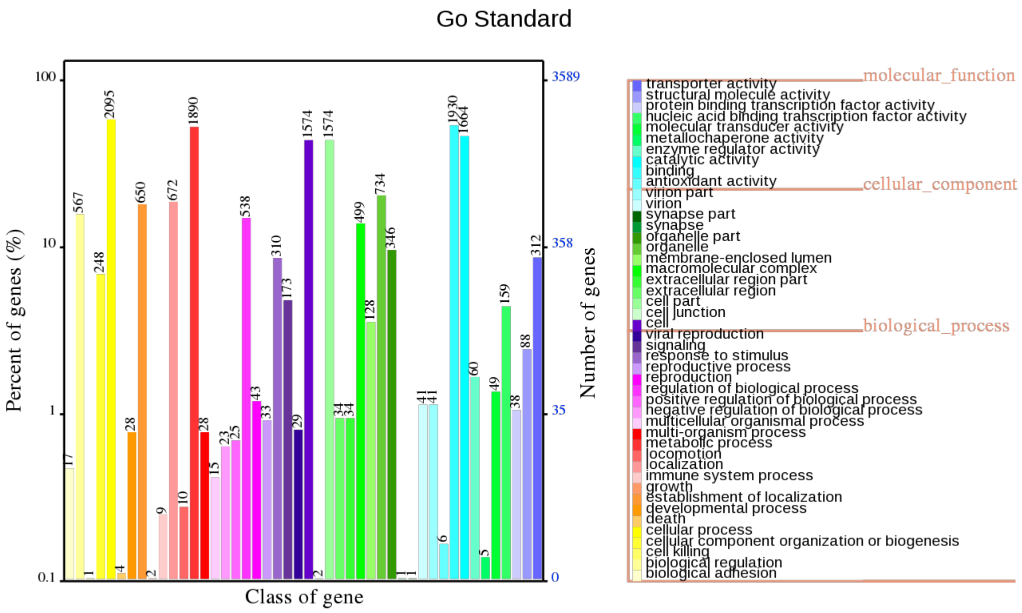

Figure S17 GO analysis of 12.1

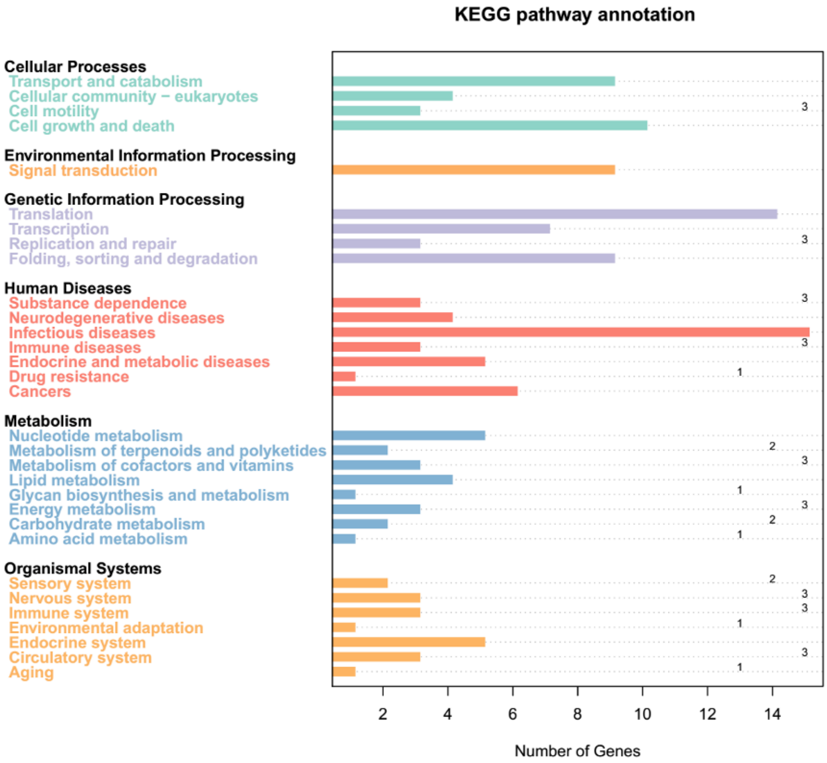

Figure S18 KEGG analysis of 1960

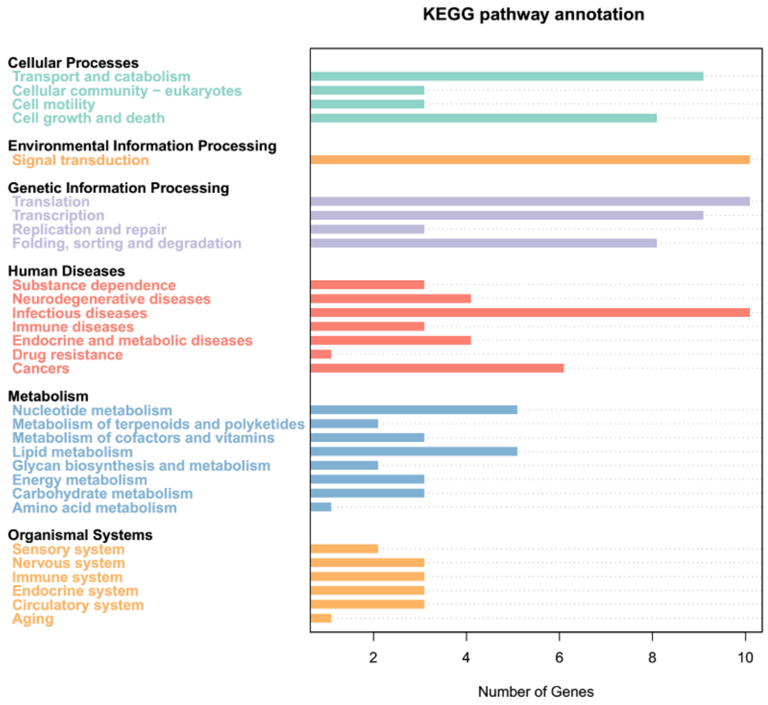

Figure S19 KEGG analysis of 31.1

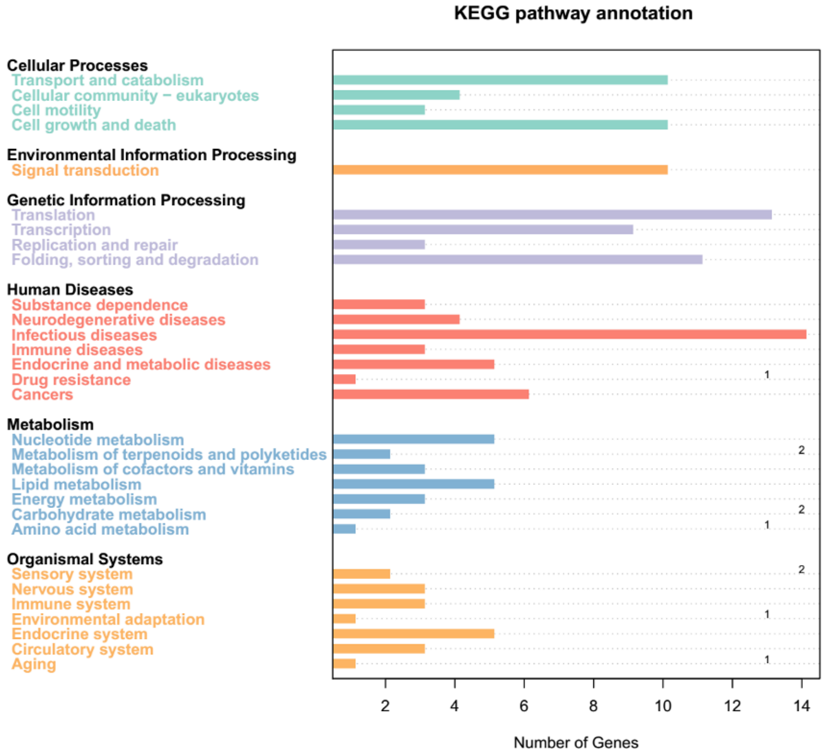

Figure S20 KEGG analysis of 12.1

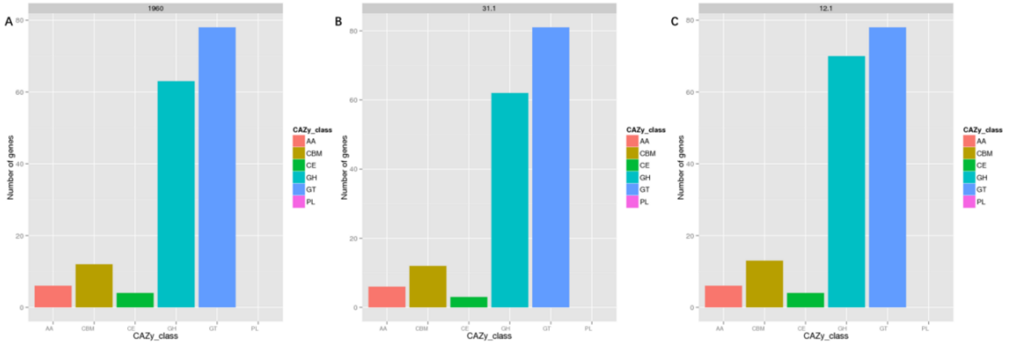

Figure S21 Analysis of CAZy

Note: A: 1960; B: 31.1; C: 12.1; The x-axis represents the classification categories of the CAZy database, the y-axis represents the number of annotated genes, and the top of the graph shows the names of each sample.

Table S11 Analysis of InDel

Sample	Insertion	Deletion	CDS_InDel
12.1	35	30	65
1960	4	5	9

Table S12 Statistics of InDel mutation

Reference Name	Sample ID	Frame-shifted	Start codon	Stop codon	Premature stop	Effect	CDS with indel	All CDS
31.1	12.1	1	0	0	1	7	9	4953
31.1	1960	1	0	0	0	2	3	4953

Table S13 Statistics of the type of InDel

Reference Name	Sample ID	Start codon In	CDS inside In	Stop codon In	Start codon Del	CDS inside Del	Stop codon Del
31.1	12.1	0	6	0	0	5	0
31.1	1960	0	2	0	0	1	0

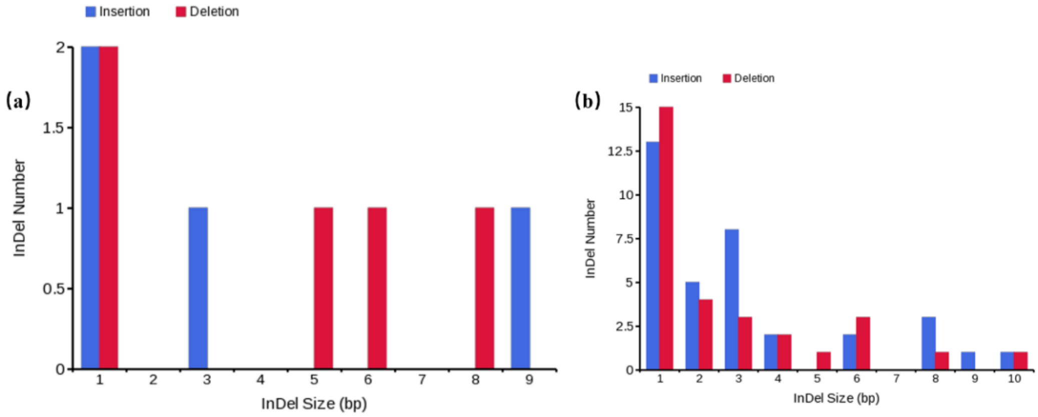

Figure S22 Analysis of InDel

Note: a: 1960; b: 12.1; The x-axis represents the length of InDels, and the y-axis represents the number of InDels。

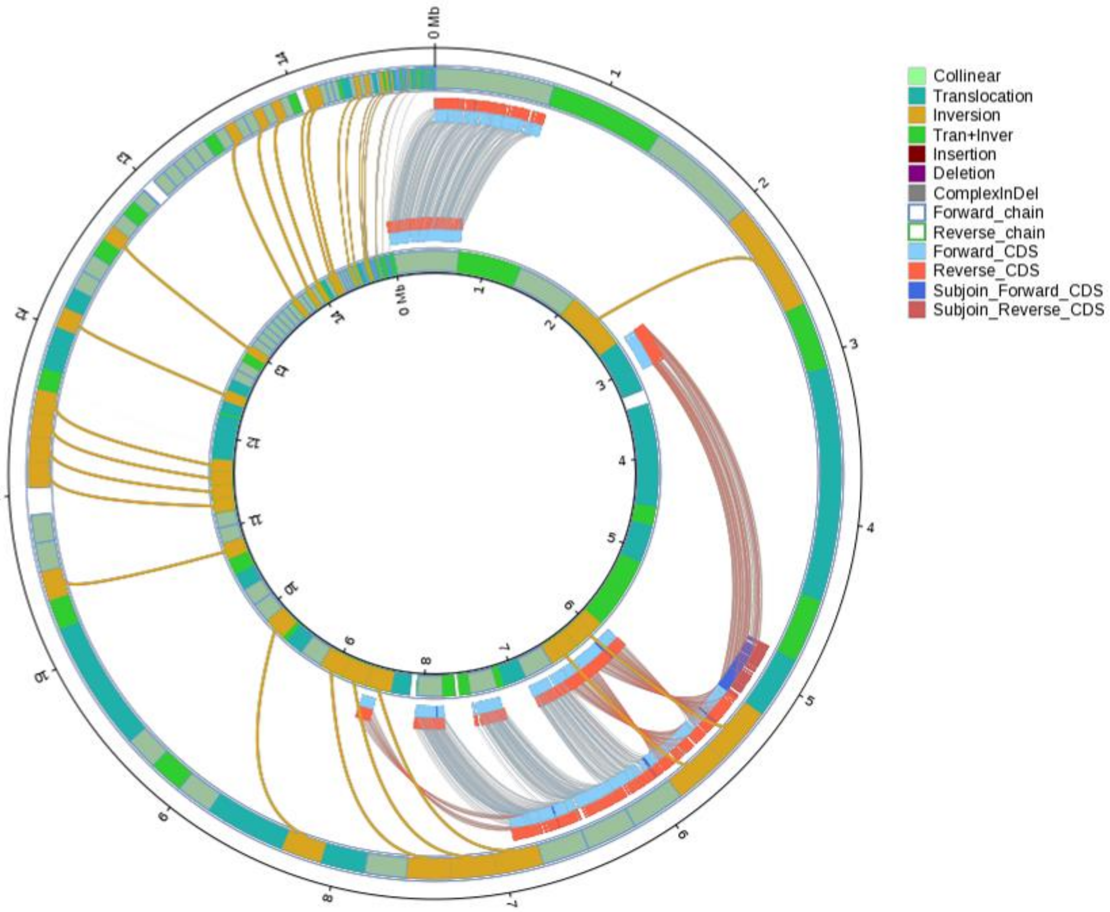

Figure S23 Analysis of SV (31.1 vs 1960)

Note：The outer circle represents the genome of the high-yield strain 31.1 (served as the reference genome), and the inner circle represents the genome of the original strain 1960. Collinear: collinearity Translocation: translocation Inversion: inversion Tran+Inver: translocation and inversion Insertion insertion (length ≥ 50 bp) Deletion: deletion (length ≥ 50 bp) ComplexInDel: complex InDel (positions correspond but cannot be aligned) Forward_chain: forward chain (gene coordinates increase in the clockwise direction) Reverse_chain: reverse chain (gene coordinates increase in the counterclockwise direction) Forward_CDS: forward CDS (CDS located on the forward chain) Reverse_CDS: reverse CDS (CDS located on the reverse chain) Subjoin_Forward_CDS: subjoin forward CDS (CDS on the forward chain of the supplementary genome) Subjoin_Reverse_CDS: subjoin reverse CDS (CDS on the reverse chain of the supplementary genome; when the paired sequence of an inserted or deleted gene shows 100% sequence identity with the gene, the CDS of this paired sequence is used as the supplementary CDS).

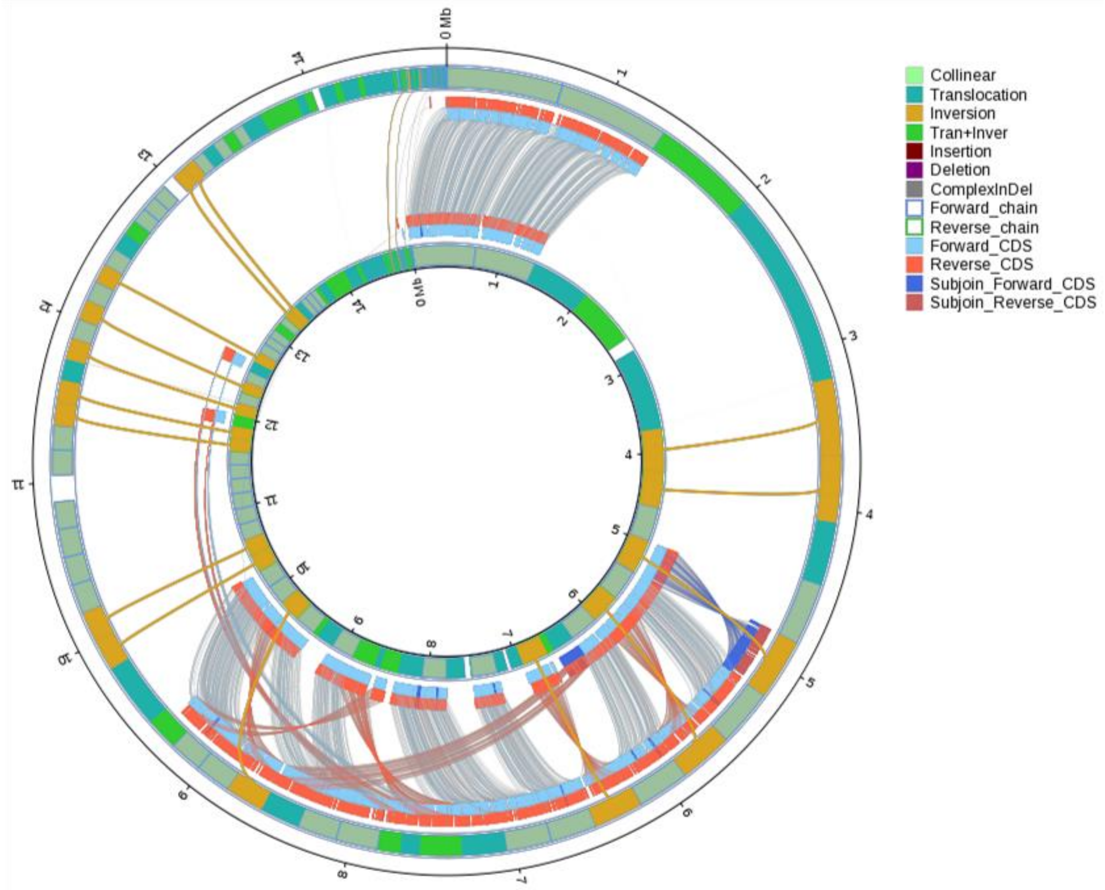

Figure S24 Analysis of SV (31.1 vs 12.1)

Note: The outer circle represents the genome of the high-yield strain 31.1 (used as the reference genome), and the inner circle represents the genome of the low-yield strain 12.1.

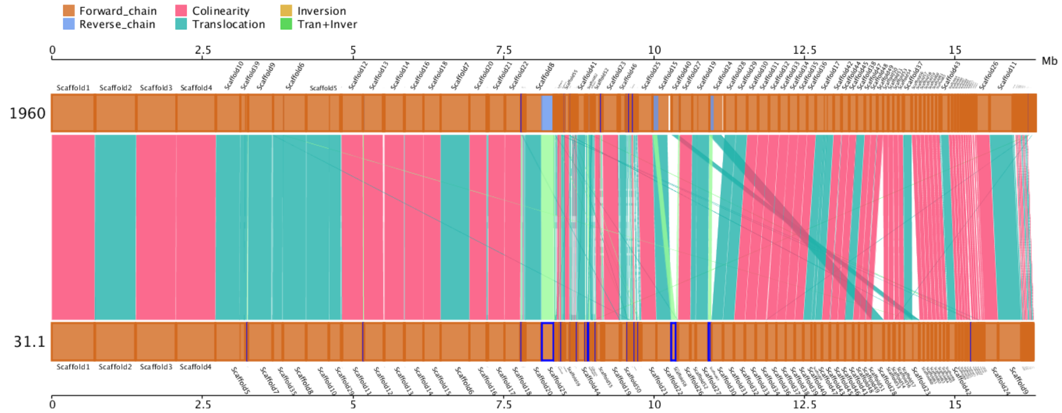

Figure S25 Collinearity analysis (31.1 vs 1960)

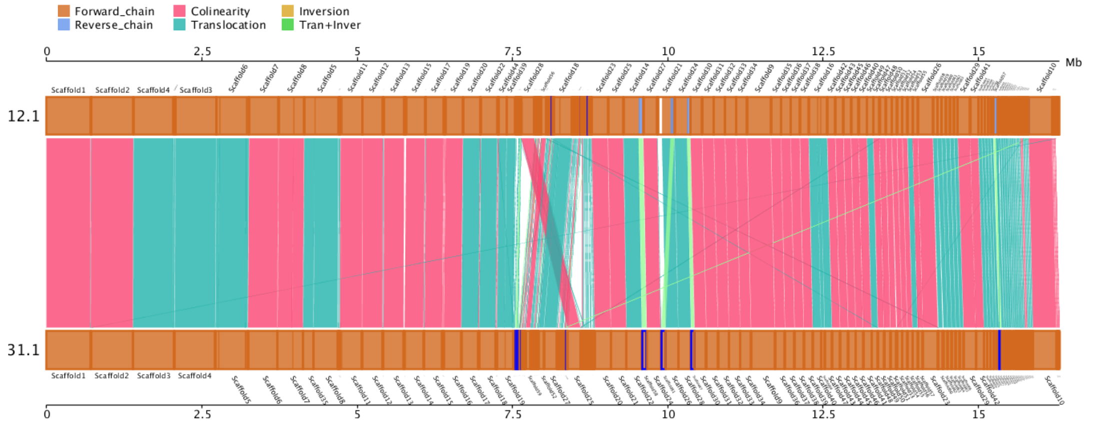

Figure S26 Collinearity analysis (31.1 vs 12.1)

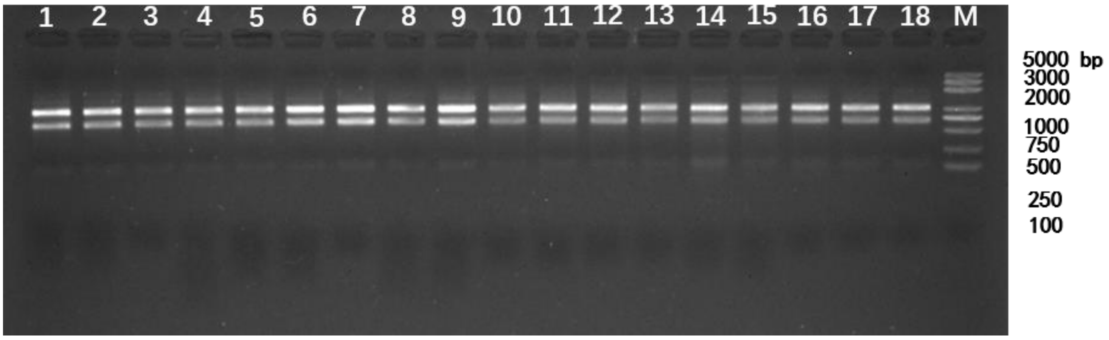

Figure S27 Total RNA of 18 samples of P. stipitis

Note: Lane: 1-3：L_12h_1, L_12h_2, L_12h_3; 4-6: H_12h_1, H_12h_2, H_12h_3; 7-9: CK_12h_1, CK_12h_2, CK_12h_3; 10-12: L_60h_1, L_60h_2, L_60h_3; 13-15: H_60h_1, H_60h_2, H_60h_3;16-18: CK_60h_1, CK_60h_2, CK_60h_3; M: Trans 2k plus Marker.

Table S14 Results of total RNA of samples

Sample Name	Concentration (ng/μL)	Volume (μL)	Total Amount (μg)	OD260/280	OD260/230	RIN	Test Conclusion
L_12h_1	2050	35.00	71.75	2.14	2.39	9.80	A
L_12h_2	2332	35.00	81.62	2.14	2.49	9.60	A
L_12h_3	2650	35.00	92.75	2.22	2.53	9.90	A
H_12h_1	2700	35.00	94.50	2.18	2.55	9.90	A
H_12h_2	2298	35.00	80.43	2.25	2.48	9.80	A
H_12h_3	3004	35.00	105.14	2.19	2.51	9.80	A
CK_12h_1	2436	35.00	85.26	2.17	2.35	9.60	A
CK_12h_2	2292	35.00	80.22	2.21	2.53	9.60	A
CK_12h_3	2646	35.00	92.61	2.25	2.53	9.50	A
L_60h_1	934	35.00	32.69	2.26	2.37	9.30	A
L_60h_2	1482	35.00	51.87	2.21	2.48	9.30	A
L_60h_3	2142	35.00	74.97	2.24	2.57	9.40	A
H_60h_1	1470	35.00	51.45	2.23	2.44	9.20	A
H_60h_2	1730	32.00	55.36	2.21	2.39	8.50	A
H_60h_3	1638	35.00	57.33	2.24	2.58	9.00	A
CK_60h_1	1748	35.00	61.18	2.24	2.53	9.40	A
CK_60h_2	1420	35.00	49.70	2.28	2.52	9.50	A
CK_60h_3	1610	35.00	56.35	2.21	2.51	9.60	A

Note: L: Low-yield strain 12.1; H: High-yield strain 31.1; CK: Wild-type strain 1960; The suffixes _1,

_2, and _3 represent 3 biological replicates; RIN: RNA Integrity Number, referring to RNA integrity value; A: The test results of the sample's total RNA are satisfactory, which meets the requirements for library construction.

Table S15 Summary for transcriptional sequencing data quality

sample	raw_reads	clean_reads	clean_bases	error_rate	Q20	Q30	GC_pct
L_12h_1	68874536	68019242	10.2G	0.02	98.28	94.85	44.75
L_12h_2	60341090	59533092	8.93G	0.02	98.22	94.67	44.98
L_12h_3	55605372	54940142	8.24G	0.02	98.19	94.64	44.94
H_12h_1	52454620	51742750	7.76G	0.02	98.30	94.90	44.92
H_12h_2	68487620	67529898	10.13G	0.02	98.10	94.39	44.95
H_12h_3	60099860	59350320	8.9G	0.02	98.23	94.73	44.95
CK_12h_1	61856658	60971974	9.15G	0.02	98.16	94.60	44.99
CK_12h_2	67493152	66606780	9.99G	0.02	98.18	94.62	43.85
CK_12h_3	56492032	55857818	8.38G	0.02	98.15	94.56	43.56
L_60h_1	60944784	60327238	9.05G	0.02	98.16	94.54	43.62
L_60h_2	45476226	44817518	6.72G	0.03	97.87	93.90	43.25
L_60h_3	49966596	49316654	7.4G	0.02	98.21	94.70	43.09
H_60h_1	41857536	41392204	6.21G	0.02	98.12	94.51	43.24
H_60h_2	52274928	51575312	7.74G	0.02	98.11	94.39	43.87
H_60h_3	61862166	61112786	9.17G	0.02	98.21	94.63	43.63
CK_60h_1	61197596	60428176	9.06G	0.02	98.19	94.59	43.81
CK_60h_2	68874536	68019242	10.2G	0.02	98.28	94.85	44.75
CK_60h_3	60341090	59533092	8.93G	0.02	98.22	94.67	44.98

Note: Sample names: L: Xylose ethanol low-yield strain 12.1; H: Xylose ethanol high-yield strain 31.1 (note: corrected for consistency with previous research context, as strain 31.1 was defined as a high-yield strain); CK: Wild-type strain 1960; The suffixes _1, _2, and _3 represent 3 biological replicates. raw_reads: Number of unprocessed reads in raw data (raw_reads); clean_reads: Number of reads in raw data after manual filtering (clean_reads); clean_bases: Number of bases in raw data after manual filtering (clean_bases = clean reads × 150 bp); error_rate: Overall error probability of the data (error_rate); Q20: Percentage of bases with a Phred score greater than 20 relative to the total number of bases (%); Q30: Percentage of bases with a Phred score greater than 30 relative to the total number of bases (%); GC_pct: Percentage of the two bases (guanine, G; cytosine, C) relative to the four total bases in clean reads (%).

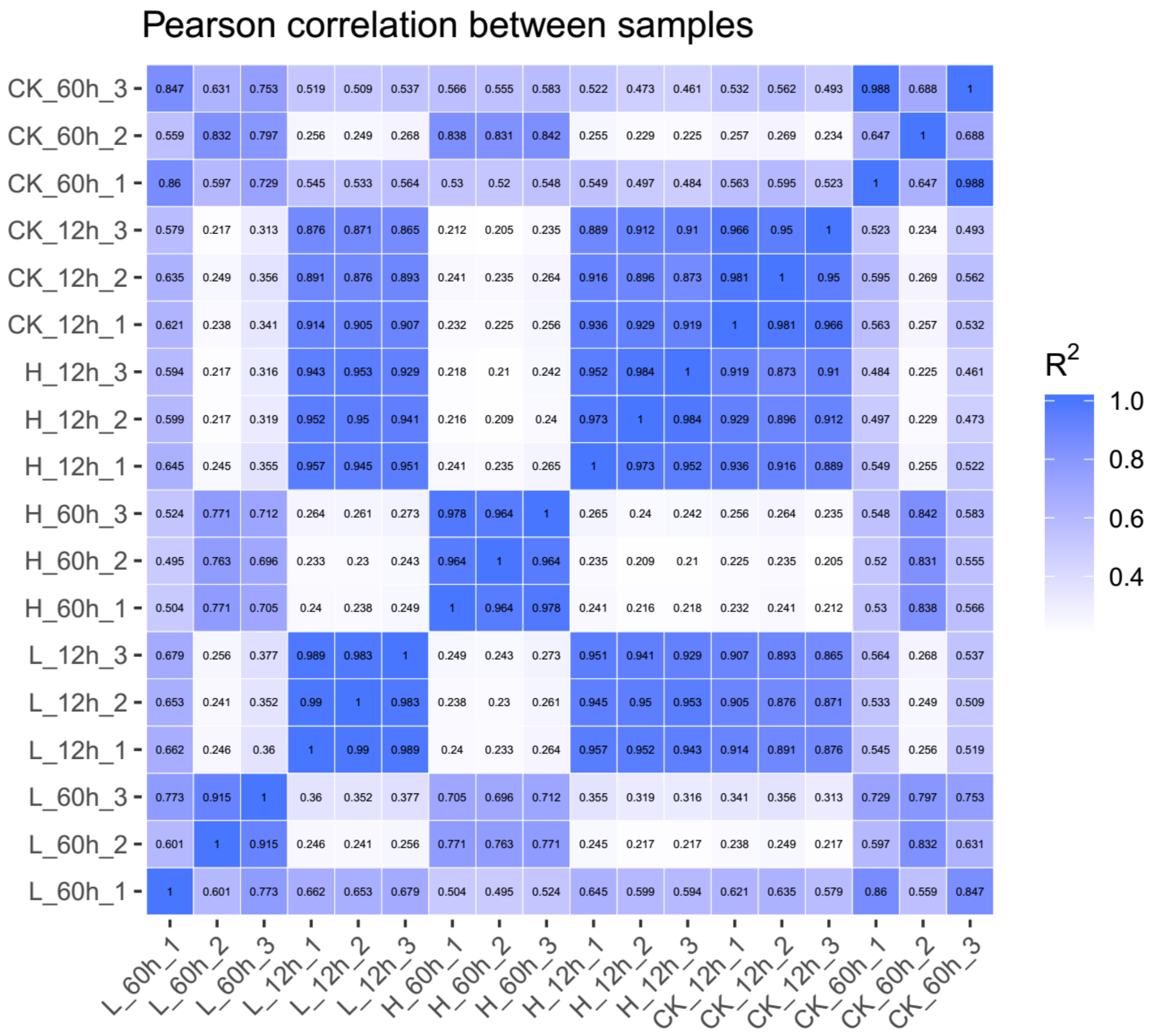

Figure S28 Analysis of the Pearson correlation coefficients of determination (R2) among three biological replicates of 6 samples

Note: CK: control strain 1960; H: xylose ethanol high-yield strain 31.1; L: xylose ethanol low-yield strain 12.1.

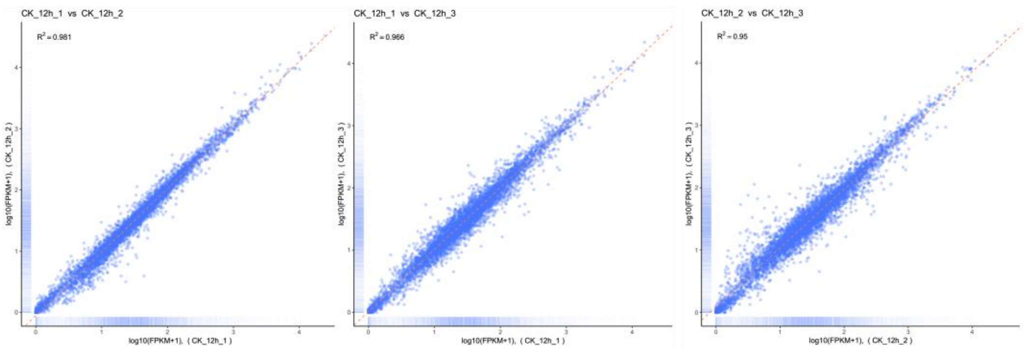

Figure S29 Analysis of the Pearson correlation coefficients of determination (R2)among three biological replicates of sample CK_12h

Note: CK: control strain 1960.

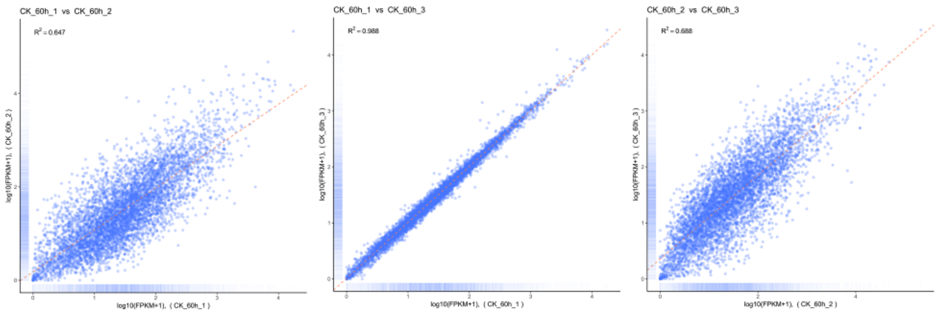

Figure S30 Analysis of the Pearson correlation coefficients of determination (R2) among three biological replicates of sample CK_60h

Note: CK: control strain 1960.

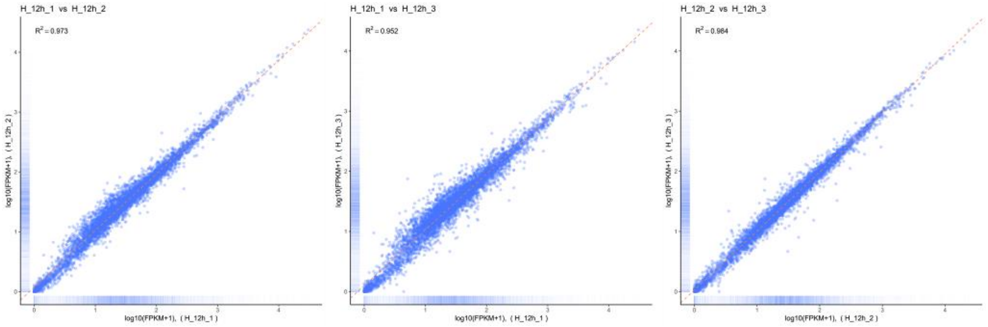

Figure S31 Analysis of the Pearson correlation coefficients of determination (R2) among three biological replicates of sample H_12h

Note: H: xylose ethanol high-yield strain 31.1.

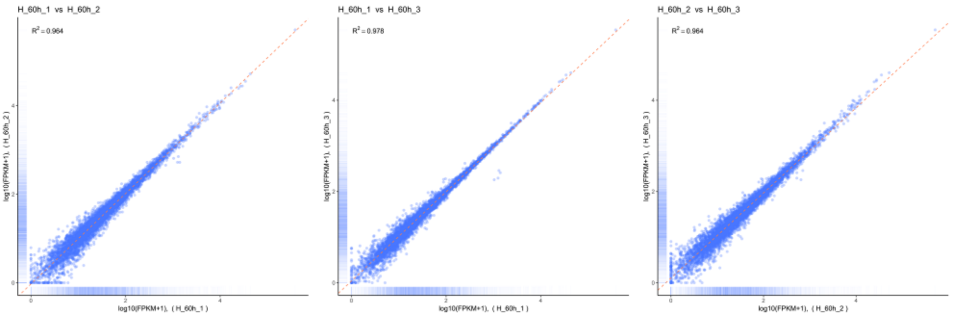

Figure S32 Analysis of the Pearson correlation coefficients of determination (R2) among three biological replicates of sample H_60h.

Note: H: xylose ethanol high-yield strain 31.1.

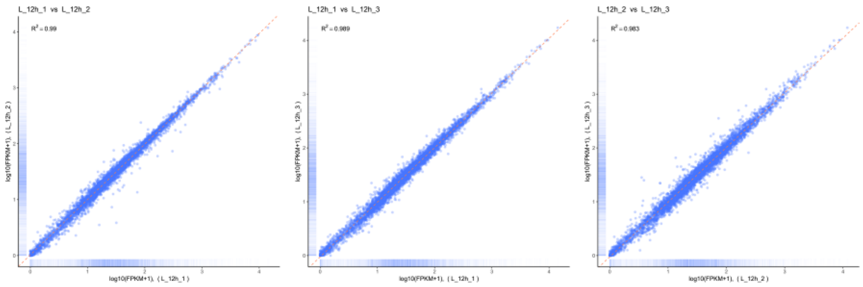

Figure S33 Analysis of the Pearson correlation coefficients of determination (R2) among three biological replicates of sample L_12h

Note: L: xylose ethanol low-yield strain 12.1.

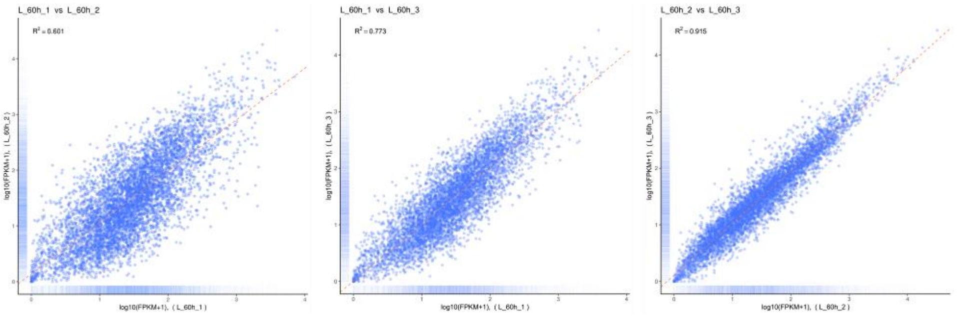

Figure S34 Analysis of the Pearson correlation coefficients of determination (R2) among three biological replicates of sample L_60h

Note: L: xylose ethanol low-yield strain 12.1.

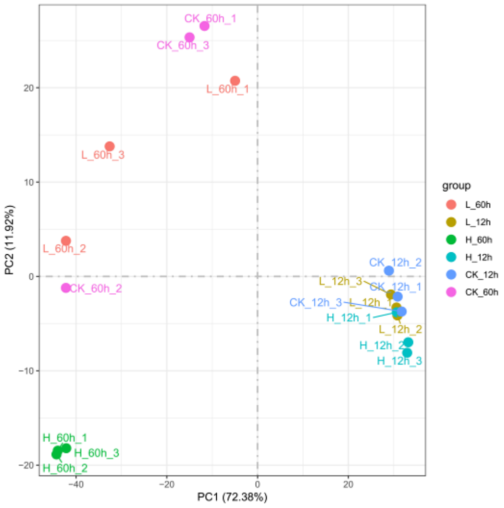

Figure S35 Principal component analysis

Note: The abscissa is the first principal component PC1,
and the ordinate is the second principal component PC2.

Table S16 Statistics of the results got by comparing with the reference genome of Pichia stipitis

Number	Sample name	Total reads	Total map	Unique map	Multi map	Positive map	Negative map	Unsplice map	Splice map
1	L_12h_1	58015096	57174836(98.55%)	56527967 (97.44%)	646869 (1.12%)	28254866 (48.7%)	28273101 (48.73%)	55716774 (96.04%)	811193 (1.4%)
2	L_12h_2	69566898	68546274(98.53%)	67737967 (97.37%)	808307 (1.16%)	33854887 (48.67%)	33883080 (48.71%)	66759101 (95.96%)	978866 (1.41%)
3	L_12h_3	68019242	67054599(98.58%)	66313326 (97.49%)	741273 (1.09%)	33145358 (48.73%)	33167968 (48.76%)	65403297 (96.15%)	910029 (1.34%)
4	H_12h_1	59533092	58731349(98.65%)	58088639 (97.57%)	642710 (1.08%)	29030352 (48.76%)	29058287 (48.81%)	57187534 (96.06%)	901105 (1.51%)
5	H_12h_2	54940142	54158502(98.58%)	53563564 (97.49%)	594938 (1.08%)	26767882 (48.72%)	26795682 (48.77%)	52809973 (96.12%)	753591 (1.37%)
6	H_12h_3	51742750	51052439(98.67%)	50500381 (97.6%)	552058 (1.07%)	25238701 (48.78%)	25261680 (48.82%)	49770327 (96.19%)	730054 (1.41%)
7	CK_12h_1	67529898	66460998(98.42%)	65713204 (97.31%)	747794 (1.11%)	32837661 (48.63%)	32875543 (48.68%)	64691232 (95.8%)	1021972 (1.51%)
8	CK_12h_2	59350320	58433073(98.45%)	57711989 (97.24%)	721084 (1.21%)	28837526 (48.59%)	28874463 (48.65%)	56813396 (95.73%)	898593 (1.51%)
9	CK_12h_3	60971974	59955460(98.33%)	59167116 (97.04%)	788344 (1.29%)	29564392 (48.49%)	29602724 (48.55%)	58230764 (95.5%)	936352 (1.54%)
10	L_60h_1	66606780	65517379(98.36%)	64505364 (96.85%)	1012015 (1.52%)	32237204 (48.4%)	32268160 (48.45%)	63814238 (95.81%)	691126 (1.04%)
11	L_60h_2	55857818	54838260(98.17%)	53802981 (96.32%)	1035279 (1.85%)	26893140 (48.15%)	26909841 (48.18%)	53387342 (95.58%)	415639 (0.74%)
12	L_60h_3	60327238	59250567(98.22%)	57908343 (95.99%)	1342224 (2.22%)	28941741 (47.97%)	28966602 (48.02%)	57401701 (95.15%)	506642 (0.84%)
13	H_60h_1	44817518	43232861(96.46%)	42741352 (95.37%)	491509 (1.1%)	21385079 (47.72%)	21356273 (47.65%)	42328873 (94.45%)	412479 (0.92%)
14	H_60h_2	49316654	46511468(94.31%)	45875705 (93.02%)	635763 (1.29%)	22948170 (46.53%)	22927535 (46.49%)	45451756 (92.16%)	423949 (0.86%)
15	H_60h_3	41392204	40284616(97.32%)	39834690 (96.24%)	449926 (1.09%)	19928813 (48.15%)	19905877 (48.09%)	39454580 (95.32%)	380110 (0.92%)
16	CK_60h_1	51575312	50759358(98.42%)	50239544 (97.41%)	519814 (1.01%)	25109493 (48.69%)	25130051 (48.72%)	49568160 (96.11%)	671384 (1.3%)
17	CK_60h_	61112786	60138506(98.41%)	59594255	544251	29795638	29798617	59088614	505641

	2			(97.52%)	(0.89%)	(48.76%)	(48.76%)	(96.69%)	(0.83%)
18	CK_60h_<br>3	60428176	59471047(98.42%)	58867959<br>(97.42%)	603088<br>(1.0%)	29428580<br>(48.7%)	29439379<br>(48.72%)	58097808<br>(96.14%)	770151<br>(1.27%)

Table S17 Statistics of DEGs of 9 groups

No.	compare	all	up	down
1	L_12h vs CK_12h	2303	1126	1177
2	H_12h vs CK_12h	1209	598	611
3	H_12h vs L_12h	1425	716	709
4	L_60h vs CK_60h	402	189	213
5	H_60h vs CK_60h	2291	991	1300
6	H_60h vs L_60h	2109	967	1142
7	CK_60h vs CK_12h	3336	1770	1566
8	L_60h vs L_12h	3824	2108	1716
9	H_60h vs H_12h	4891	2455	2436

Note: CK: Wild-type strain 1960; H: Xylose ethanol high-yield strain 31.1; L: Xylose ethanol low-yield strain 12.1; 12 h, 60 h: 12 h of fermentation, 60 h of fermentation; Statistical threshold (Threshold): All follow the criteria of DESeq2 padj < 0.05 and |log2FoldChange| > 0; Compare: Comparisons between different groups; All: Total number of differential genes; Up: Number of up-regulated genes; Down: Number of down-regulated genes.

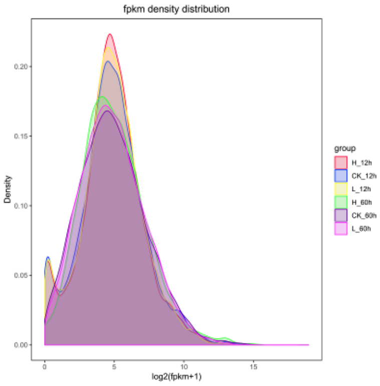

Figure S36 Gene density of samples

Note: The abscissa is log2(FPKM+1), and the ordinate is the gene density.

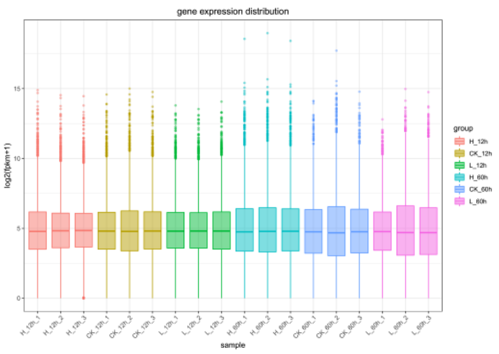

Figure S37 Box diagram of gene expression quantity distribution of samples

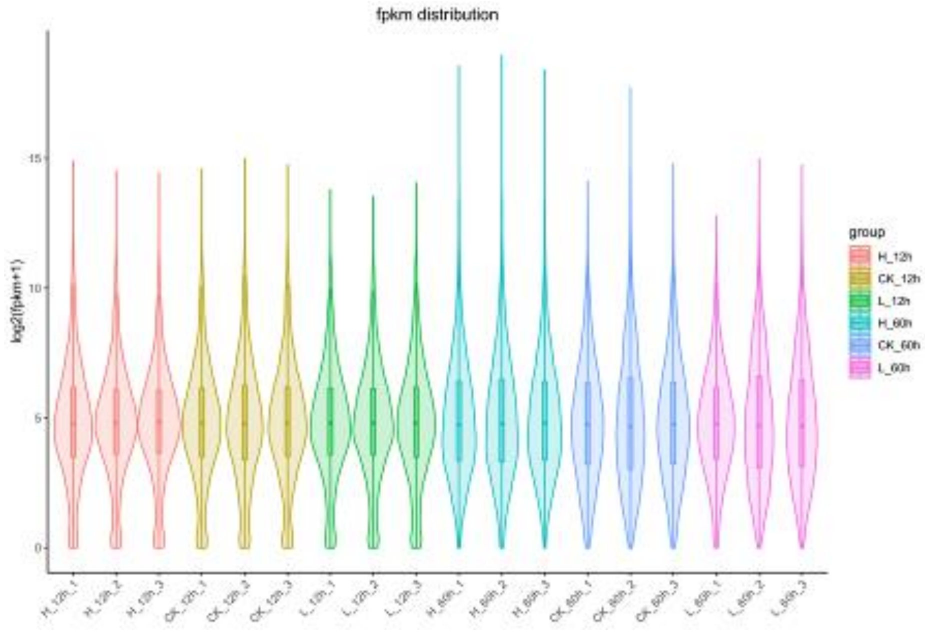

Figure S38 Violin chart

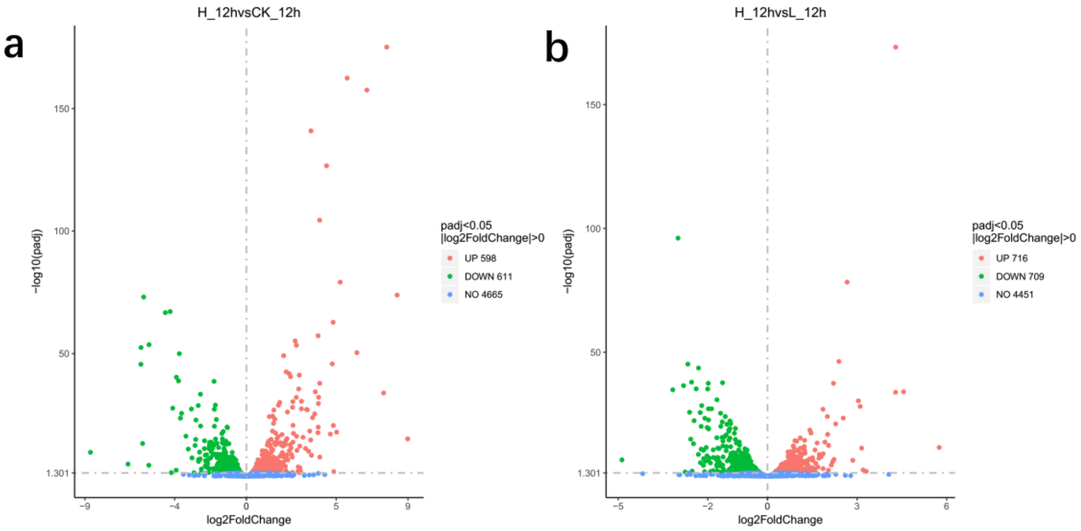

Figure S39 Differential analysis of gene expression between H_12h and CK_12h (a); H_12h and L_12h (b)

Note: H: high-yield strain 31.1; CK: the control strain 1960; L: low-yield strain 12.1.

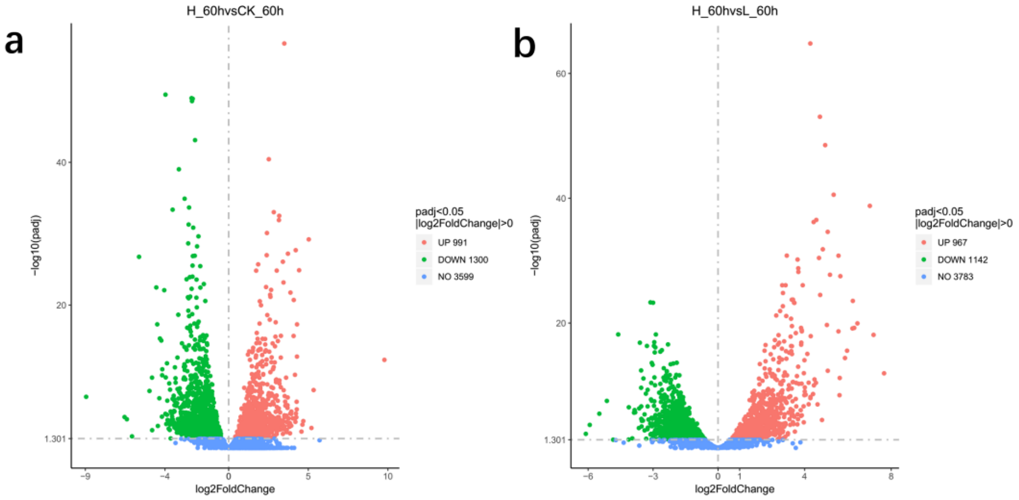

Figure S40 Differential analysis of gene expression between H_60h and CK_60h (a); H_60h and L_60h (b)

Note: H: high-yield strain 31.1; CK: the control strain 1960; L: low-yield strain 12.1.

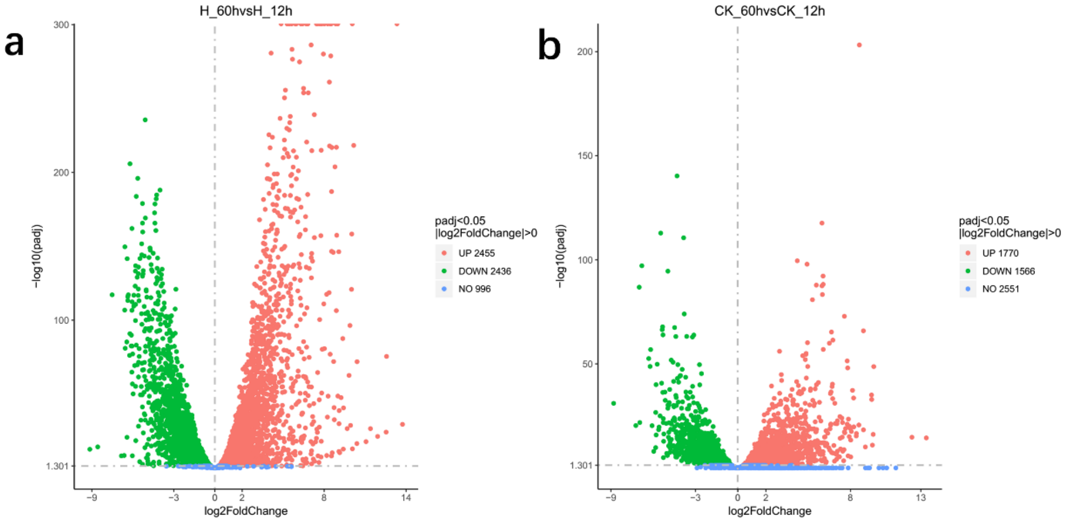

Figure S41 Differential analysis of gene expression between H_60h and H_12h (a); CK_60h and CK_12h (b)

Note: H: high-yield strain 31.1; CK: the control strain 1960.

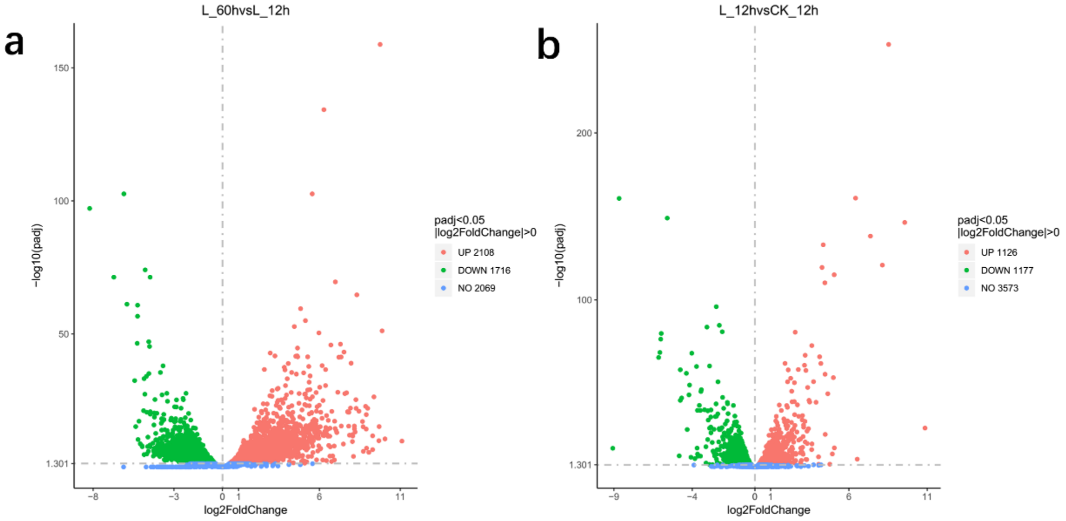

Figure S42 Differential analysis of gene expression between L_60h and L_12h (a); L_12h and CK_12h (b)

Note: L: low-yield strain 12.1; CK: the control strain 1960.

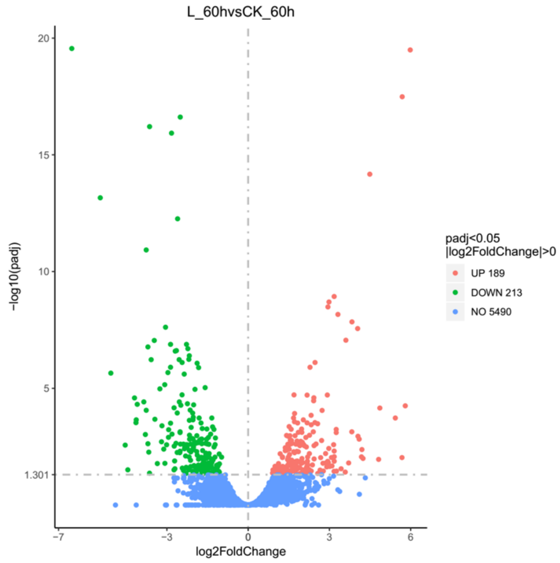

Figure S43 Differential analysis of gene expression between L_60h and CK_60h

Note: L: low-yield strain 12.1; CK: the control strain 1960.

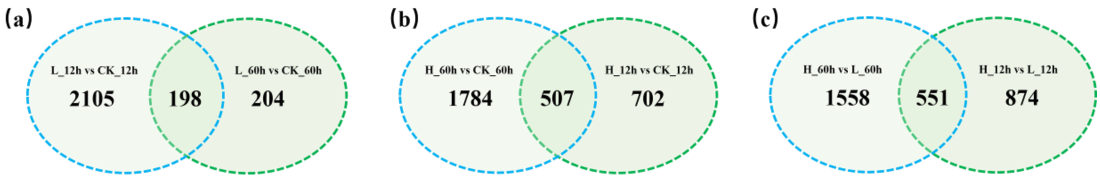

Figure S44 Overlapping of different genes in different comparison combinations

Note: a: L_12h vs CK_12h, L_60h vs CK_60h; b: H_12h vs CK_12h, H_60h vs CK_60h; c: H_12h vs L_12h, H_60h vs L_60h; H: high-yield strain 31.1; CK: the control strain 1960; L: low-yield strain12.1.

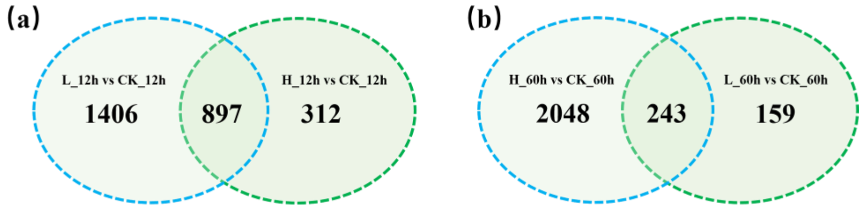

Figure S45 Overlapping of different genes in different comparison combinations

Note: a: L_12h vs CK_12h, H_12h vs CK_12h; b: L_60h vs CK_60h, H_60h vs CK_60h; H: high-yield strain 31.1; CK: the control strain 1960; L: low-yield strain 12.1.

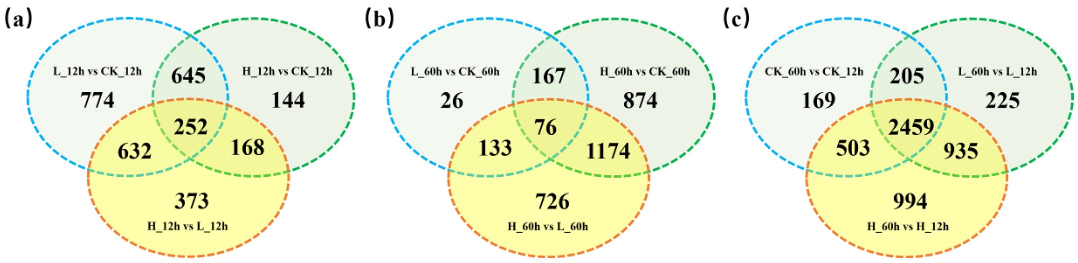

Figure S46 Overlapping of different genes in different comparison combinations

Note: a: L_12h vs CK_12h, H_12h vs CK_12h, H_12h vs L_12h; b: L_60h vs CK_60h, H_60h vs CK_60h, H_60h vs L_60h; c: CK_60h vs CK_12h, H_60h vs H_12h, L_60h vs L_12h; H: high-yield strain 31.1; CK: the control strain 1960; L: low-yield strain 12.1.

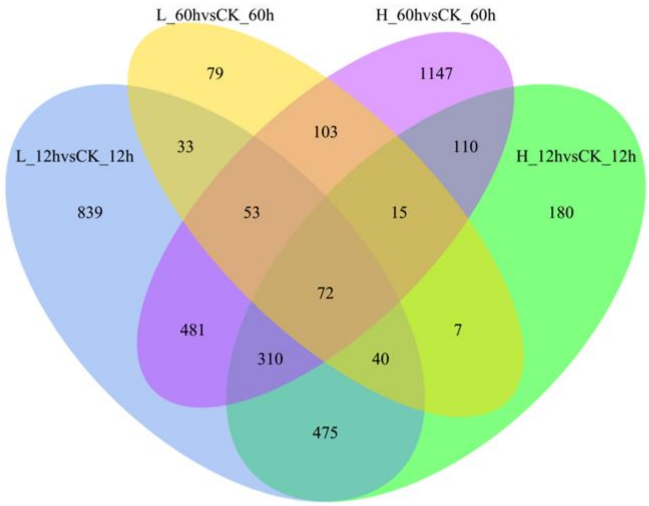

Figure S47 Overlapping of different genes in different comparison combinations

Note: L_12h vs CK_12h, L_60h vs CK_60h, H_12h vs CK_12h, H_60h vs CK_60h; H: high-yield strain 31.1; CK: the control strain 1960; L: low-yield strain 12.1.

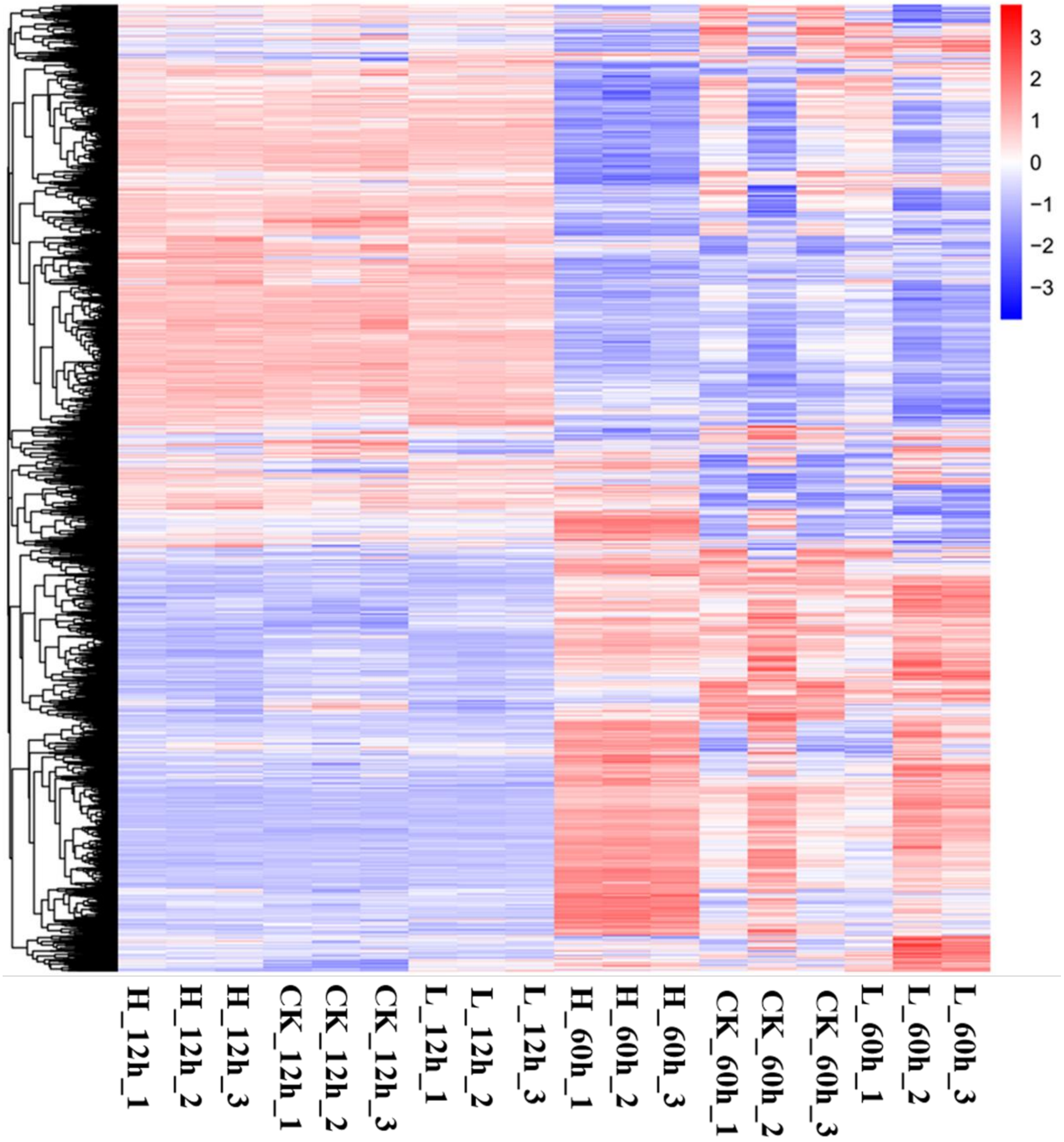

Figure S48 Cluster heat map of DEGs

Note: H: high-yield strain 31.1; CK: the control strain 1960; L: low-yield strain 12.1; In the figure, the abscissa is the sample name, and the ordinate is the normalized value of the FPKM of DEGs.The redder the color, the higher the expression, the bluer the expression, the lower the expression.

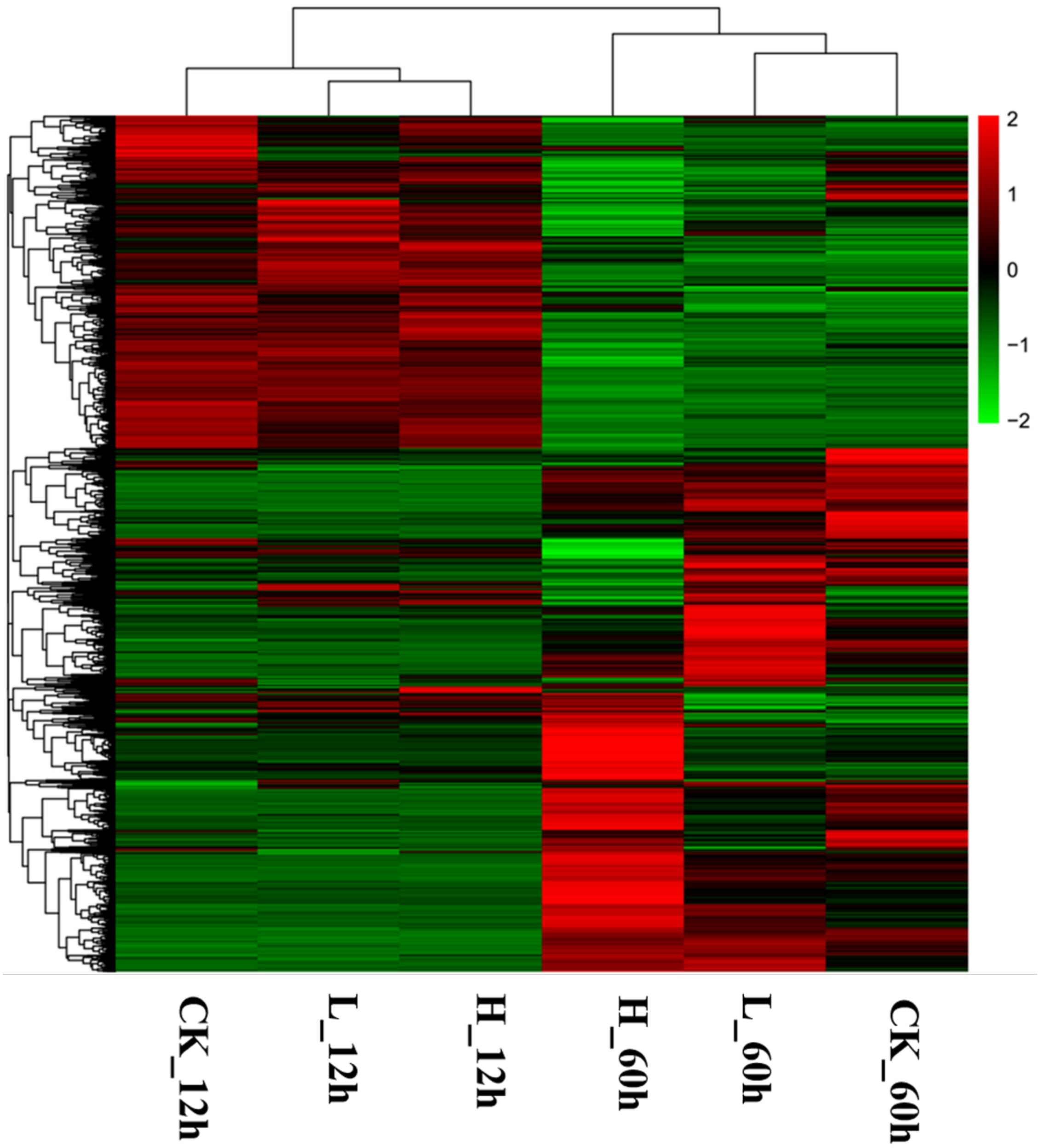

Figure S49 Cluster heat map of DEGs

Note: H: high-yield strain 31.1; CK: the control strain 1960; L: low-yield strain 12.1; In the figure, the abscissa is the sample name, and the ordinate is the normalized value of the FPKM of DEGs.The

redder the color, the higher the expression, the greener the expression, the lower the expression.
